# Neural Assimilation and Accommodation in Trilinguals: An fMRI Study at 7 T

**DOI:** 10.1002/hbm.70620

**Published:** 2026-07-31

**Authors:** Dinh Ha Duy Thuy, Naoya Oishi, Thai Akasaka, Tomohisa Okada, Takashi Hanakawa

**Affiliations:** ^1^ Human Brain Research Center, Graduate School of Medicine Kyoto University Kyoto Japan; ^2^ Support Unit for Functional Magnetic Resonance Imaging RIKEN Center for Brain Science Wako Japan; ^3^ Department of Integrated Neuroanatomy and Neuroimaging, Graduate School of Medicine Kyoto University Kyoto Japan

**Keywords:** accommodation, assimilation, fMRI, linguistic distance, multilingualism, representational similarity analysis (RSA)

## Abstract

Understanding how multiple languages are represented in the multilingual brain is critical to theories of multilingual assimilation and accommodation. Whether a second language (L2) recruits native‐like networks (assimilation) or engages additional neural resources (accommodation) depends partly on the linguistic distance between L1 and L2. However, it remains unclear whether multilinguals can flexibly modulate assimilation and accommodation mechanisms when dealing with different L2 systems. Using 7 T‐fMRI, we investigated brain activation patterns during a covert picture‐naming task involving one‐back phonological retrieval in 15 native Chinese speakers highly proficient in Japanese and English. Whole‐brain univariate analysis revealed a common fronto‐parieto‐occipital network across all three languages (Chinese, Japanese, and English), supporting a shared core language system. However, the Japanese task elicited greater activation in the left lingual and middle occipital gyri, suggesting increased implicit visual‐lexical demands. Representational Similarity Analysis (RSA) showed greater pattern similarity between Chinese and English than between Chinese and Japanese, indicating differential neural representation across L2 languages. VOI‐based RSA showed a trend toward correlations between Japanese proficiency and representational similarity in the executive regions (pars triangularis and anterior cingulate gyrus), although these correlations did not reach statistical significance. These findings demonstrated that the trilingual brain relied on both assimilation and accommodation, with the level of each modulated by differences in linguistic structure and language experience. Language with a distinct writing system (English) seemed to be assimilated into the dominant L1 Chinese neural framework, whereas orthographically related language (Japanese) rather drove neural accommodation, probably due to script‐driven interference. This study highlighted multi‐level neural adaptation processes underlying multilingual lexical processing and provided a neurocognitive basis for tailor‐made educational strategies considering each student's linguistic background.

## Graphical abstract

Using brain activation and representational similarity analysis at 7 T‐MRI, this study demonstrated that the trilingual brain flexibly modulated neural assimilation and accommodation. While Chinese and English shared highly similar neural representations, Japanese engaged distinct patterns, highlighting script‐driven effects on multilingual neural organization.

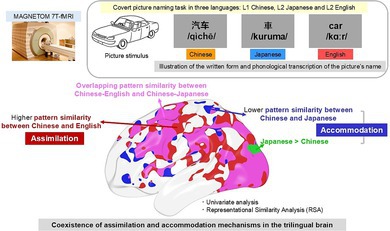

## 1 Introduction

Understanding how multiple languages are represented and processed in the multilingual brain remains a central question in neurolinguistics. One key issue is whether second languages (L2s), particularly those with different writing systems, are processed in the brain in the same way as the native language (L1) or engage distinct neural pathways. Recent research on bilingual language processing has suggested two different theories to explain how L2 information is integrated and processed in the brain: assimilation and accommodation models (Abutalebi and Green 2016; Perani and Abutalebi 2005; Perfetti and Liu 2005; Perfetti et al. 2007). Assimilation refers to the integration of new L2 linguistic input into existing L1‐based neural schemas, whereas accommodation involves the recruitment of additional neural resources to handle L2‐specific features that differ significantly from L1.

Whether an L2 language is assimilated or accommodated should depend on multiple factors, such as age of acquisition (AoA), language proficiency, learning context, and linguistic distance. AoA has been regarded as a determinant; early exposure to L2 tends to favor assimilation, while late acquisition increases the likelihood of accommodation (Berken et al. 2016; Bloch et al. 2009; Hernandez and Li 2007; Kim et al. 1997; Liu et al. 2020). The proficiency level of L2 also plays a crucial role. Highly proficient L2 learners show more L1‐like activation patterns, reflecting a shift from accommodation to assimilation over time, while less proficient L2 learners show accommodation patterns (Cao et al. 2013; Consonni et al. 2013; Perani et al. 1998). Learning context, whether formal or immersive, can also shape how L2 is integrated into the L1 existing language network (Costa and Sebastián‐Gallés 2014; Yang et al. 2015).

Additionally, linguistic distance, defined as the degree of structural similarity between L1 and L2 orthography, phonology, and syntax (Chai and Bao 2022; Chiswick and Miller 2005), has consistently been indicated as a major factor. When L2 shares structural similarities with L1, the brain tends to assimilate L2 processing into pre‐existing L1 neural networks (Kim et al. 2016). Studies comparing language processing within the same script family, such as Spanish‐English or Italian‐English bilinguals, revealed highly overlapping activation patterns in the left peri‐Sylvian language regions, suggesting assimilation (Illes et al. 1999; Perani et al. 1998). In contrast, when L2 differs substantially in orthographic and phonological structure from L1, reduced representational similarity between L2 and L1 and the recruitment of additional neural regions not typically active in L1 processing have been reported (Buchweitz et al. 2009; Nelson et al. 2009; Wang et al. 2020). For example, Buchweitz et al. (2009) found that, in native Japanese speakers, L2 English reading more extensively recruited left angular and inferior frontal regions than L1 Japanese reading, reflecting greater reliance on grapheme‐to‐phoneme conversion mechanisms. Similarly, studies using multivariate pattern analysis (MVPA) and Representational Similarity Analysis (RSA) have demonstrated that orthographically distant L2 exhibited lower neural representational similarity with L1, indicating novel mappings through accommodation (Dong et al. 2021). However, this set of rules cannot fully cover the real‐world complexity of cross‐language relationships. For instance, although both Chinese and Japanese Kanji are logographic, they differ markedly in their phonological characteristics. In Chinese, each character typically has a single, context‐invariant pronunciation. In contrast, Japanese Kanji often have multiple readings that vary depending on the grammatical role or context in which they appear (Hashimoto et al. 2020; Thuy et al. 2004). Previous neuroimaging studies have shown that native Chinese speakers elicited stronger activation of the left lateral prefrontal cortex during phonological processing of L2 Japanese Kanji than during L1 Chinese phonological processing. This likely reflected higher cognitive demand and increased accommodation, despite L2 shared orthographic features with L1 (Lin et al. 2024). Another study reported that when native Chinese speakers processed orthographically distant L2 English, English was unexpectedly easy to assimilate due to its systematic phoneme‐to‐letter correspondences (Chee et al. 1999; Nelson et al. 2009). Together, these mixed findings on linguistic distance impact highlighted the need for more nuanced investigations into how multiple linguistic dimensions of distinct writing systems jointly shape L2 neural representations.

Another limitation of previous studies is that most presented written words as visual stimuli for comparisons between L1 and L2. This raises a methodological concern because visual word input may introduce bias due to inherent differences in script familiarity between writing systems, such as logographic versus alphabetic scripts. Such differences could confound cross‐linguistic comparisons by engaging distinct visual word recognition mechanisms (Bolger et al. 2005; Kim et al. 2020; Tan et al. 2005). To mitigate these confounds and isolate language‐specific effects, nonverbal stimuli, such as pictures, may offer a promising alternative. In fact, picture naming tasks allow us to probe the semantic and phonological processes associated with lexical retrieval, making them suitable to investigate language mapping across languages with diverse writing systems (Ala‐Salomäki et al. 2021; Indefrey and Levelt 2004; Levelt et al. 1999; Li et al. 2013). When comparing between overt and covert picture‐naming tasks, a covert task is preferable because it involves less head motion. Covert picture‐naming tasks can elicit activation in core language regions, including the left inferior frontal gyrus, the middle temporal gyrus, and the supplementary motor areas, consistent with findings from overt tasks (Abutalebi and Green 2007; Price 2012).

Finally, because most prior neuroimaging work has focused on bilinguals, who learned only a single L2, our understanding of how the brain organizes multiple non‐native languages remains limited. Studying only one L2 limits our ability to explore whether assimilation and accommodation can coexist within the same multilingual brain, especially when different L2s have varying degrees of linguistic distance from L1. This question can be directly addressed in those speakers whose L1 is logographic (e.g., Chinese) and acquire both an orthographically similar L2 (e.g., Japanese Kanji) and an orthographically distant L2 (e.g., English).

Here, using a covert picture‐naming task, the present fMRI study examined the neural representation of proficient trilinguals who mastered Chinese as L1 and two orthographically distinct non‐native languages (English and Japanese). This design allowed us to determine whether the emergence of neural assimilation and/or accommodation patterns depends mainly on cross‐language distance by comparing both brain activation and pattern similarity across the three language conditions. We also assessed whether additional factors, such as AoA and proficiency level of L2s, modulate these patterns by including them as covariates in the analysis.

## 2 Methods

### 2.1 Participants

This study included 15 healthy native Chinese speakers (ages 21–30 years; 6 males and 9 females) with no prior neurological or psychiatric disorders. The number of participants was determined based on a within‐subject design which is sensitivity for detecting language‐related effects compared with between‐subject designs. As previously reported, assuming a medium effect size (*f* = 0.25), an alpha of 0.05 and statistical power of 0.80, a repeated‐measures analysis of variance (ANOVA) required approximately 17 participants (Dong et al. 2021). The present sample of 15 participants was slightly below this estimate but was consistent with the sample sizes reported in previous within‐subject fMRI studies of bilingual and multilingual language processing (Chen et al. 2026; Hernandez et al. 2000; LeBel et al. 2023; Xue et al. 2004).

Study criteria included high proficiency in both Japanese and English. Proficiency in Japanese was proven by N1 certification of the Japanese Language Proficiency Test (JLPT) and/or graduation from, or participation in, undergraduate programs carried out in Japanese universities. Four of the fifteen participants graduated from a Japanese university but have not yet taken the JLPT (thus, N1 scores unavailable). English proficiency was confirmed by English standard test scores meeting university‐level requirements, such as TOEFL ≥ 80, IELTS ≥ 6.0, or TOEIC ≥ 750. The TOEFL and IELTS scores were converted to equivalent TOEIC scores based on a conversion table (https://www.se.tmu.ac.jp/prospect/conv‐non‐bio_rigaku.pdf). The average AoA was 17.7 for Japanese (range 13–24 years old), while 6.6 for English (range 3–12 years old). There was a statistically significant difference between them (t(14) = 13.46, *p* = 0.0001, Cohen's *d* = 3.47).

All participants were right‐handed, as assessed by the Edinburgh Handedness Inventory (Oldfield 1971), and had normal or corrected‐to‐normal visual acuity. All participants provided written informed consent in accordance with the protocol approved by the ethics committee of the Kyoto University Graduate School of Medicine.

### 2.2 Experimental Materials and Tasks

Stimuli consisted of 240 black‐and‐white line drawings of common objects, selected from a standardized picture database (Nishimoto et al. 2005). These objects were reported to have high familiarity and name agreement among Japanese native speakers. The pictures included both natural and man‐made objects. A white background image (blank) was used for null stimulus trial, yielding two stimulus lists, each containing 160 stimuli (120 object pictures + 40 blanks).

To assess cross‐language naming consistency for the full stimulus set used in this study, a separate behavioral naming experiment was conducted in an independent sample of 10 native Chinese speakers who were highly proficient in Japanese and English. None of them participated in the fMRI experiment. This assessment was conducted to confirm the consistency of picture naming across the Chinese‐Japanese‐English trilingual populations rather than to serve as a formal normative study of the stimuli. The detailed procedure for computing naming agreement is provided in the Supporting Information (Table S5).

Before the fMRI session, all participants underwent a training session outside the scanner to familiarize them with the task and to ensure that they understood the instructions. In this pre‐scan training, participants practiced the picture‐naming task overtly in Chinese, Japanese, and English, using 30 stimuli, a subset of the entire stimulus list. The training confirmed that participants could accurately and fluently retrieve the correct names for each picture in all three languages, verifying both their L2 proficiency and naming congruency.

**FIGURE 1:**
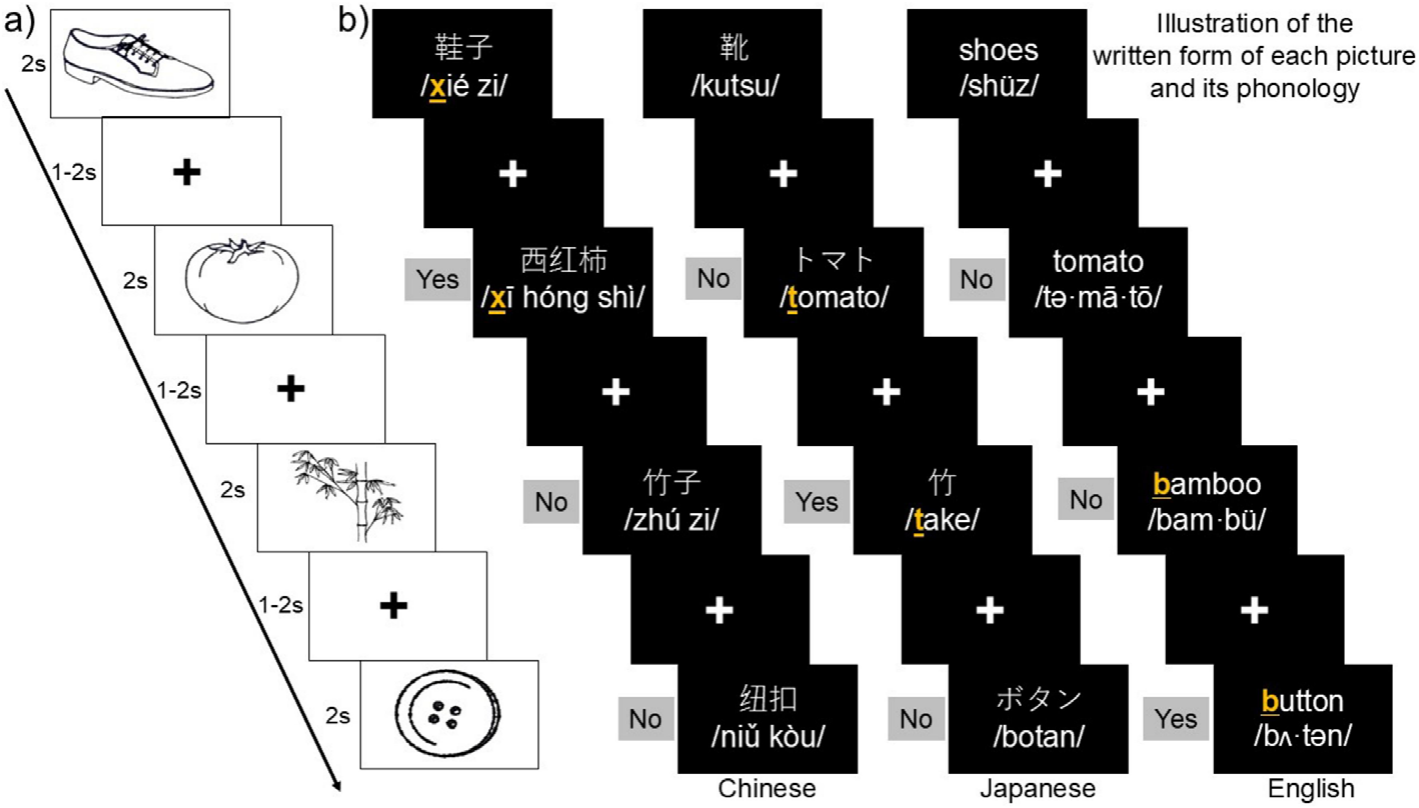
Experimental materials and task design (a) Examples of picture stimuli used in the experiment; (b) Illustration of the written forms and mental phonological transcriptions of their names used in the covert picture‐naming involving phonological one‐back task. Participants pressed the “Yes” button when the initial grapheme onset of the presented picture's name matched that of the preceding item, and the “No” button when it did not. Grapheme onsets were defined by Pinyin for Chinese, Romaji for Japanese, and the alphabet for English.

During fMRI sessions, participants performed a covert picture‐naming task, combined with a phonological one‐back task, in Chinese, Japanese, and English (a one‐back covert picture‐naming task). Each participant completed six fMRI runs; each language condition was assigned to each run and repeated twice using two different stimulus lists, such that each list was assigned to one of the two runs for that language. This ensured that the same items were not repeated within the same language condition. The order of task runs across the three language conditions was counterbalanced across participants. Each run consisted of the 160 stimuli presented in random order, lasting about 10 min. Each stimulus trial was presented for 2 s, interleaved with visual fixation trials that varied randomly from 1 to 2 s (Figure 1a). Participants covertly named an object displayed on the monitor and decided whether the initial grapheme onset of its name matched that of the preceding item (i.e., phonological one‐back task). To enable cross‐script comparability across three languages, grapheme onsets were defined using Pinyin for Chinese, Romaji for Japanese, and the English alphabet. Participants provided their responses by pressing response buttons with their index and middle fingers for “yes” and “no” answers, respectively (Figure 1b). Participants were instructed to respond as quickly and as correctly as possible. Stimuli presentation, timing, and behavioral data collection were controlled by E‐Prime 2.0.

### 2.3 fMRI Data Acquisition

Scanning was performed on a MAGNETOM 7 T whole‐body MRI scanner (Siemens Healthineers, Erlangen, Germany) using a single‐channel transmitter and 32‐channel receiver head coil (Nova Medical, MA, USA). Participants lay supine in the MRI scanner. Head motion was minimized using high‐permittivity dielectric pads placed bilaterally at the zygomatic regions, which also aimed to improve B1+ inhomogeneity.

For functional volumes, a multiband blood oxygen level‐dependent (BOLD) sensitive T‐2*‐weighted gradient‐echo (GRE) echo planar imaging (EPI) sequence (research prototype sequence) (Moeller et al. 2010) was acquired with the following parameters: repetition time (TR) = 1000 ms, echo time (TE) = 22 ms, flip angle (FA) = 45°, bandwidth (BW) = 1924 Hz/pixel, multiband acceleration factor (MB) = 5, generalized auto‐calibrating partially parallel acquisition (GRAPPA) factor = 2, spatial resolution = isotropic 1.6 mm, 85 slices. The EPI images in the two fMRI runs for the same language condition were acquired with opposite phase‐encoding directions: anterior‐to‐posterior (AP) and posterior‐to‐anterior (PA). After acquiring the functional images, a whole‐brain magnetization‐prepared two rapid acquisition gradient echo MP2RAGE (Marques et al. 2010) anatomical images were acquired as follows: TR = 6000 ms, TE = 2.9 ms, inversion time (TI)1 = 800 ms and TI2 = 2700 ms, FA1 = 4° and FA2 = 5°, spatial resolution = isotropic 0.7 mm, 256 slices. Uniform and denoised images acquired using MP2RAGE (O'Brien et al. 2014) were used in this study because their contrast was similar to that of MPRAGE images without a noisy background.

### 2.4 Data Analysis

#### 2.4.1 Behavioral Data Analysis

A one‐way repeated‐measures ANOVA was performed separately for response time (RT) and accuracy to examine the effect of language (i.e., L1 Chinese (CN), L2 Japanese (JA), and L2 English (EN)). When a significant main effect was found, post hoc pairwise comparisons were conducted between all language pairs (CN‐JA, CN‐EN, and JA‐EN) using Bonferroni‐corrected tests to control for multiple comparisons.

To ensure that a speed‐accuracy trade‐off did not drive the observed differences in RT across language conditions, we calculated the inverse efficiency score (IES) by dividing the mean RT by the accuracy for each condition and participant (Bruyer and Brysbaert 2011; Dong et al. 2021). Additionally, to examine whether behavioral performance was associated with individual differences in AoA and proficiency of the two L2s, correlation analyses were conducted between individual participants' behavioral performance (RT and accuracy) and their corresponding AoA and proficiency scores. Furthermore, to determine whether the behavioral performance difference between the two L2s was affected by their AoA differences, correlations between the RT and accuracy differences between JA and EN, and the difference in their AoA were also tested.

#### 2.4.2 fMRI Data Preprocessing

The fMRI data were preprocessed using a combination of FSL (FMRIB Software Library v6.0.7.1) and the Computational Anatomy Toolbox (CAT12) toolbox (version r2137; http://www.neuro.uni‐jena.de/cat/) implemented in SPM12 (Wellcome Trust Centre for Neuroimaging, London, UK). First, motion correction was conducted using FSL's MCFLIRT tool, realigning each volume to the middle time point (Jenkinson et al. 2002). To correct for susceptibility‐induced distortions, a B0‐unwarping step using FSL's TOPUP (Andersson et al. 2003) was applied using a pair of EPI images with opposite phase‐encoding directions. Then, EPI images underwent bias‐field correction to reduce intensity non‐uniformities. Non‐brain tissues were removed using FSL‐anat. The resulting motion‐corrected and distortion‐corrected images were used for subsequent co‐registration. The functional images were co‐registered to the corresponding MP2RAGE image using the FSL boundary‐based registration (BBR) tool. The MP2RAGE image was spatially normalized to the standard Montreal Neurological Institute (MNI) template using geodesic shooting registration (Ashburner and Friston 2011) with CAT12 for segmentation into gray matter, white matter, and cerebrospinal fluid (CSF) and for individual deformation fields to Montreal Neurological Institute (MNI) space. These deformation fields were later used to normalize the functional images to the MNI152 standard space. Finally, for the whole‐brain univariate analysis, the normalized functional images were spatially smoothed with a 5 mm Full Width at Half Maximum (FWHM) Gaussian kernel using SPM12. To preserve fine‐grained voxel‐wise activation patterns, spatial smoothing was not applied for fMRI data used for Representational Similarity Analysis (RSA).

#### 2.4.3 Univariate fMRI Analysis

A two‐stage analysis was performed using SPM 12 (Wellcome Trust Center for Neuroimaging, UCL, London). At the first level, a general linear model (GLM) was applied to model task‐related BOLD signals. Boxcar regressors for task events were convolved using the canonical hemodynamic response functions. All six fMRI runs (two per language) were included in each subject's design matrix to evaluate the linear contrast and obtain contrast images. Then the contrast images were entered for second‐level analysis. At the second level, brain activation associated with each language condition was detected using a one‐sample *t*‐test. We also conducted a one‐way repeated‐measures ANOVA and *post hoc* comparisons to evaluate the main effects of languages and pairwise differences across the three language conditions. To ensure that the observed activation differences between language conditions were not attributed to variation in participants' L2 backgrounds, the AoA and proficiency scores of each L2 were incorporated as covariates in the second‐level analysis. Unless otherwise stated, statistical maps were thresholded at *p* < 0.001 voxel‐wise (uncorrected) and cluster *p* < 0.05 with family‐wise error (FWE)‐corrected for multiple comparison (Chumbley et al. 2010).

#### 2.4.4 Representational Similarity Analysis (RSA)

To investigate the similarity of neural activity patterns across the three language conditions, we also conducted RSA (Kriegeskorte et al. 2006, 2008). Both a whole‐brain searchlight approach and predefined volumes of interest (VOIs) were conducted. Whole‐brain searchlight RSA (Xue et al. 2013) was performed across the whole‐brain voxels to identify regions where local multi‐voxel patterns encode information that distinguishes or overlaps between the three language conditions. To this end, a 4 mm radius spherical searchlight was centered at each voxel to extract activation patterns for the CN, JA, and EN conditions separately. Cross‐language pattern similarity, that is, between Chinese and Japanese (CN‐JA), Chinese and English (CN‐EN), and Japanese and English (JA‐EN), was quantified using Pearson correlation coefficients, which were subsequently converted into Fisher's *z*‐scores. The searchlight method was implemented in each participant's run and then averaged across the two runs. Individual participants' similarity maps were spatially smoothed with a 5‐mm FWHM and then submitted to a group‐level random‐effects analysis. The AoA and proficiency scores of each L2 were incorporated as covariates in this second‐level analysis. We also examined the effect of orthographic distance on cross‐language pattern similarity by applying a paired *t*‐test to compare pattern similarity for the CN‐EN and CN‐JA.

VOI‐based RSA was also conducted to assess the extent to which neural representations in the key brain regions related to lexical retrieval and picture naming differ between the three language conditions. We predefined eight left hemispheric VOIs that are consistently involved in picture naming, lexical retrieval, and executive control of non‐native languages (Ala‐Salomäki et al. 2021; Crinion et al. 2006; Okada et al. 2000; Price 2012). These VOIs included the fusiform gyrus (FG), posterior inferior temporal gyrus (PITG), pars triangularis (PT), pars opercularis (PO), supramarginal gyrus (SMG), superior parietal lobe (SPL), anterior cingulate cortex (ACC), and head of caudate nucleus (CN). VOIs were anatomically defined based on MNI coordinates from the Harvard‐Oxford probabilistic atlas (Maximal Probability threshold: 25%) in the FSL standard atlases. Similar to the whole‐brain RSA, within each VOI, cross‐language pattern similarity between CN‐EN, CN‐JA, and JA‐EN was calculated using Pearson correlation analysis followed by Fisher's *z*‐score transformation. A one‐way repeated‐measures ANOVA was performed on these *z*‐scores to test the effects of cross‐language pattern similarity in these VOIs. Furthermore, to examine whether this pattern similarity was associated with individual differences in AoA and proficiency of the two L2s, correlation analyses were also conducted.

To assess the robustness of the primary findings, leave‐one‐out (LOO) sensitivity analyses were performed by iteratively excluding one participant at a time and repeating both the univariate analysis and RSA across all 15 iterations. The stability of the peak coordinates, *t*‐values, cluster sizes, and Fisher's *z*‐scores was evaluated across iterations to determine whether the reported effects were driven by any individual participant.

### 2.5 Statistics

All correlation analyses in this study were performed using Pearson's correlation. To examine the independent contributions of AoA and proficiency of L2, additional partial correlation analyses were also performed by controlling for proficiency when evaluating AoA effects and by controlling for AoA when evaluating proficiency effects. The correlation between AoA and proficiency in each L2 language was also examined to assess the extent of shared variance between the two variables. Unless otherwise stated, the Kolmogorov–Smirnov test was conducted to confirm that the input variables were normally distributed. For the repeated‐measures ANOVA, the Greenhouse–Geisser correction was applied when the assumption of sphericity was not met. Effect sizes are reported for all inferential tests. For paired *t*‐tests and post hoc comparisons, Cohen's d was computed using the standard deviation of the difference scores, appropriate for within‐subject designs, and partial eta squared (partial η^2^) was reported for the repeated‐measures ANOVA.

## 3 Results

### 3.1 Behavioral Results

**FIGURE 2:**
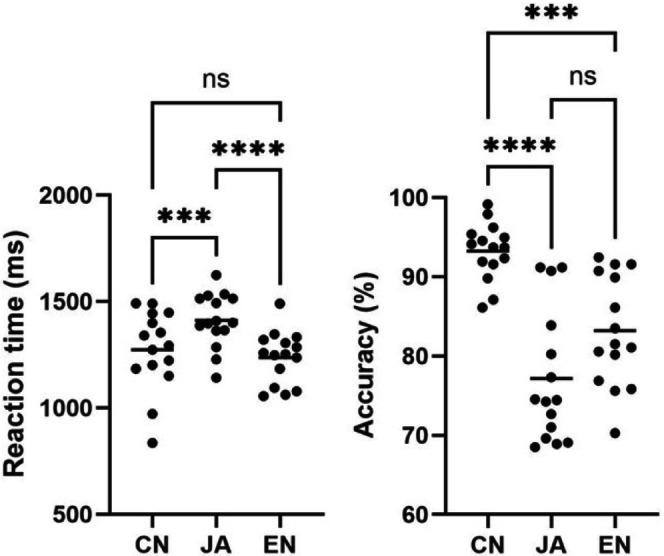
Behavioral performance. Mean response time (RT) and accuracy. Picture naming in the Japanese language showed longest RTs and lowest accuracy, indicating higher processing demand. Significant effects are indicated with asterisks (***p < 0.001, ****p < 0.0001).

A significant main effect of languages was observed for both RT [F(1.95, 27.23) = 23.60, *p* < 0.001, partial η^2^ = 0.63] and accuracy [F(1.723, 24.12) = 33.66, *p* < 0.001, partial η^2^ = 0.71] (Figure 2). Post hoc comparisons showed that RTs were slowest in JA (1412.25 ms), which were significantly longer than both CN (1273.39 ms, *p* < 0.001, Cohen's d (dz) = 1.29) and EN (1237.04 ms, *p* < 0.0001, dz. = 1.84). No difference in RTs was observed between CN and EN (*p* = 0.42, dz. = 0.33). Accuracy was highest for CN (93.24%), which was significantly greater than both JA (77.17%, *p* < 0.0001, dz. = 2.32) and EN (83.20%, *p* < 0.001, dz. = 1.48). By contrast, accuracy for JA and EN did not differ (*p* = 0.054, dz. = 0.66).

**FIGURE 3:**
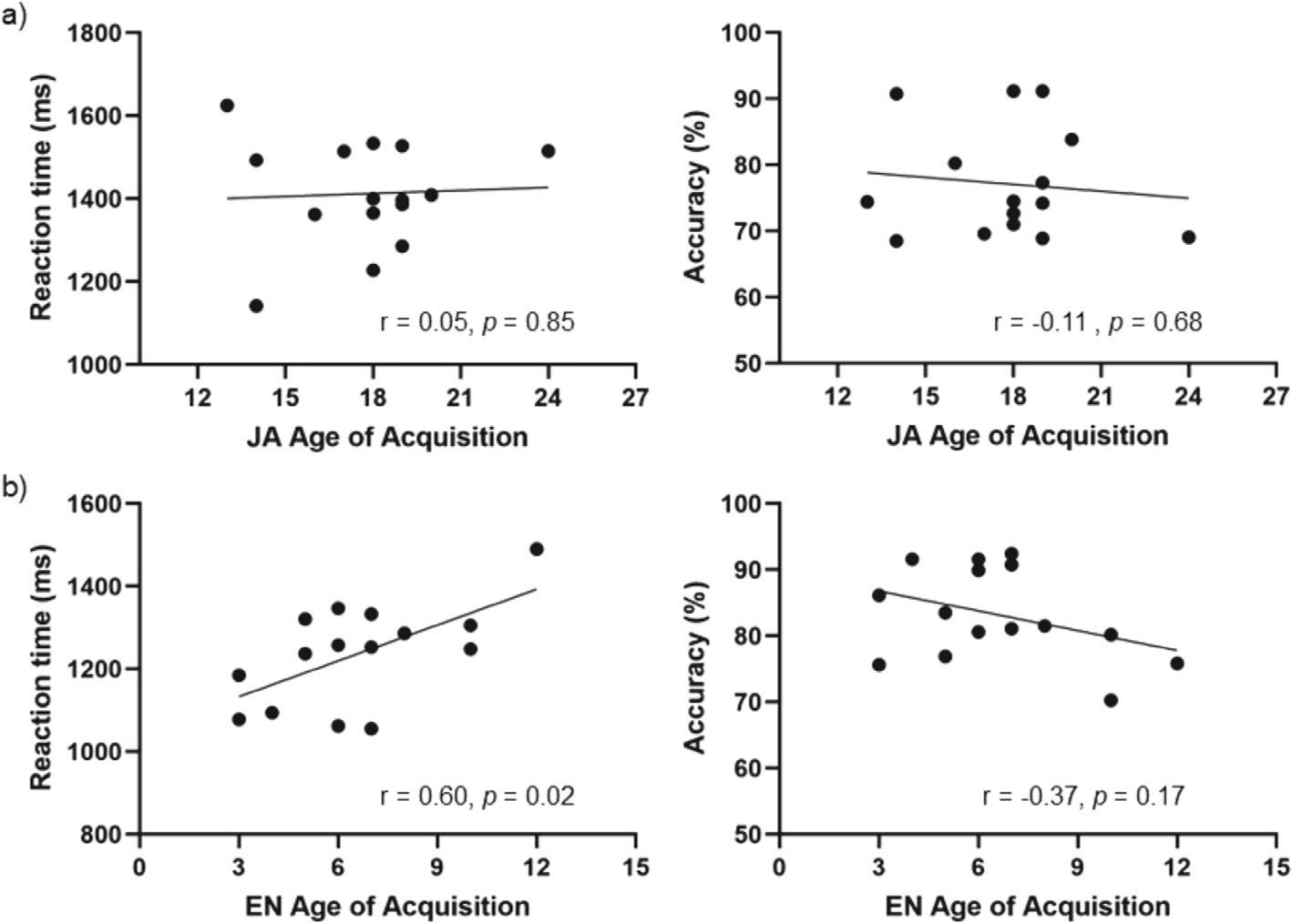
Correlation between behavioral performance and Age of Acquisition of L2s. Correlations between RT (on the left) and accuracy (on the right) and AoA of Japanese (a) and English (b). A significant positive correlation was observed between AoA and RT in the English condition. Other correlations did not reach statistical significance.

**FIGURE 4:**
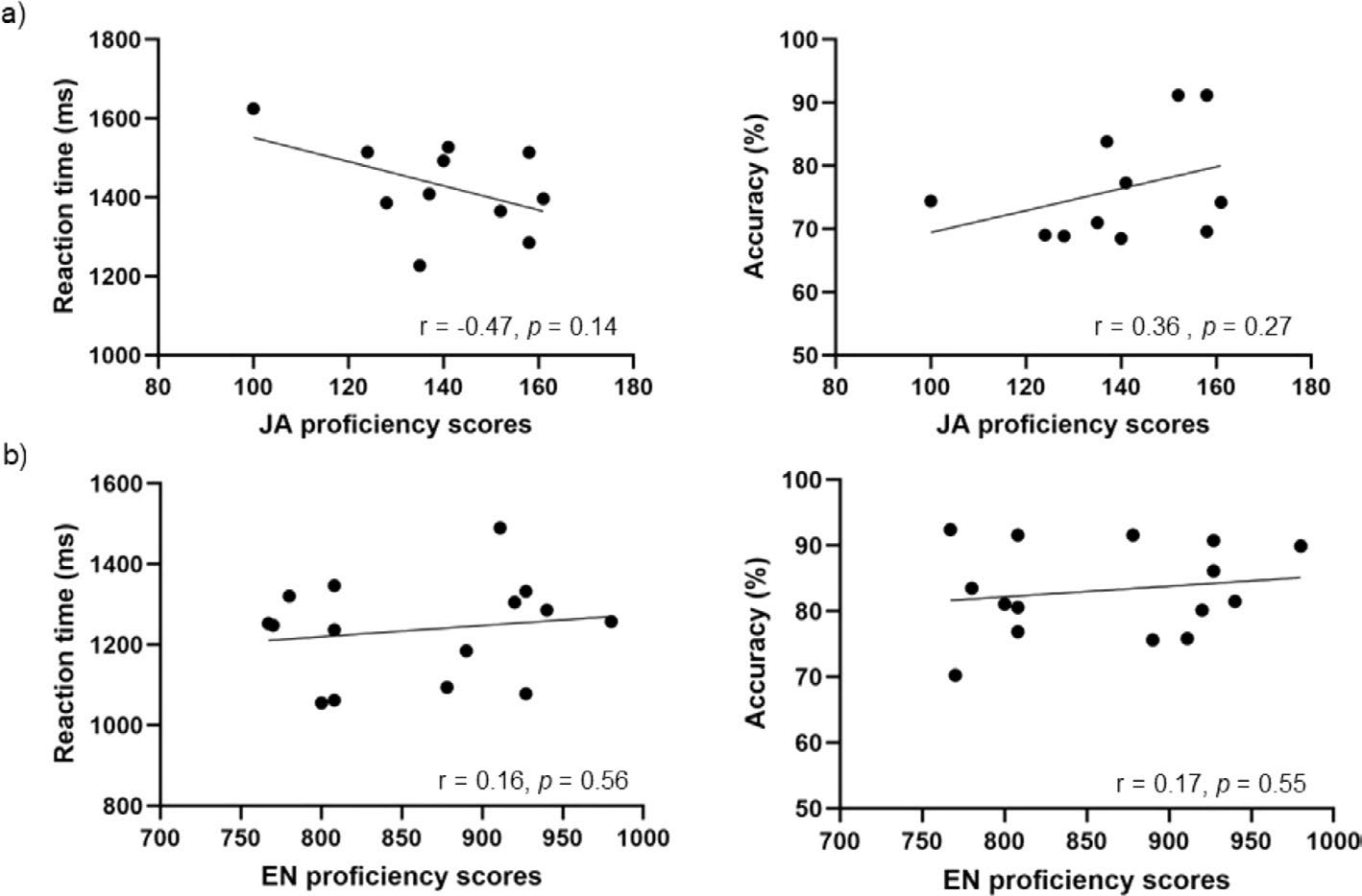
Correlation between behavioral performance and L2s proficiency Correlations of RT and accuracy with Japanese (a) and English (b) proficiency scores. None reached statistical significance (all *p* > 0.1). RT showed a moderate negative correlation with Japanese proficiency scores (*r* = −0.47, *p* = 0.14).

Because the JA condition yielded the longest RT and the lowest accuracy, it was excluded from the IES analysis. EN showed significantly higher IES values than CN (t(14) = 2.83, *p* = 0.01, dz. = 0.69), indicating poorer overall efficiency. The correlation analysis between behavioral performance and L2 AoA revealed a positive correlation between RT and EN AoA (*r* = 0.6, *p* = 0.02). No other correlations were statistically significant (Figure 3). For L2 JA, there was a trend toward correlation between proficiency scores and RT (*r* = −0.47, *p* = 0.14) and accuracy (*r* = 0.36, *p* = 0.27) (Figure 4a). No correlations were observed between EN proficiency scores and behavioral performance (Figure 4b). The correlation between the differences in RT and accuracy between JA and EN and the difference in their AoA did not reach statistical significance (data not shown).

### 3.2 fMRI Results of Univariate Analysis

**FIGURE 5:**
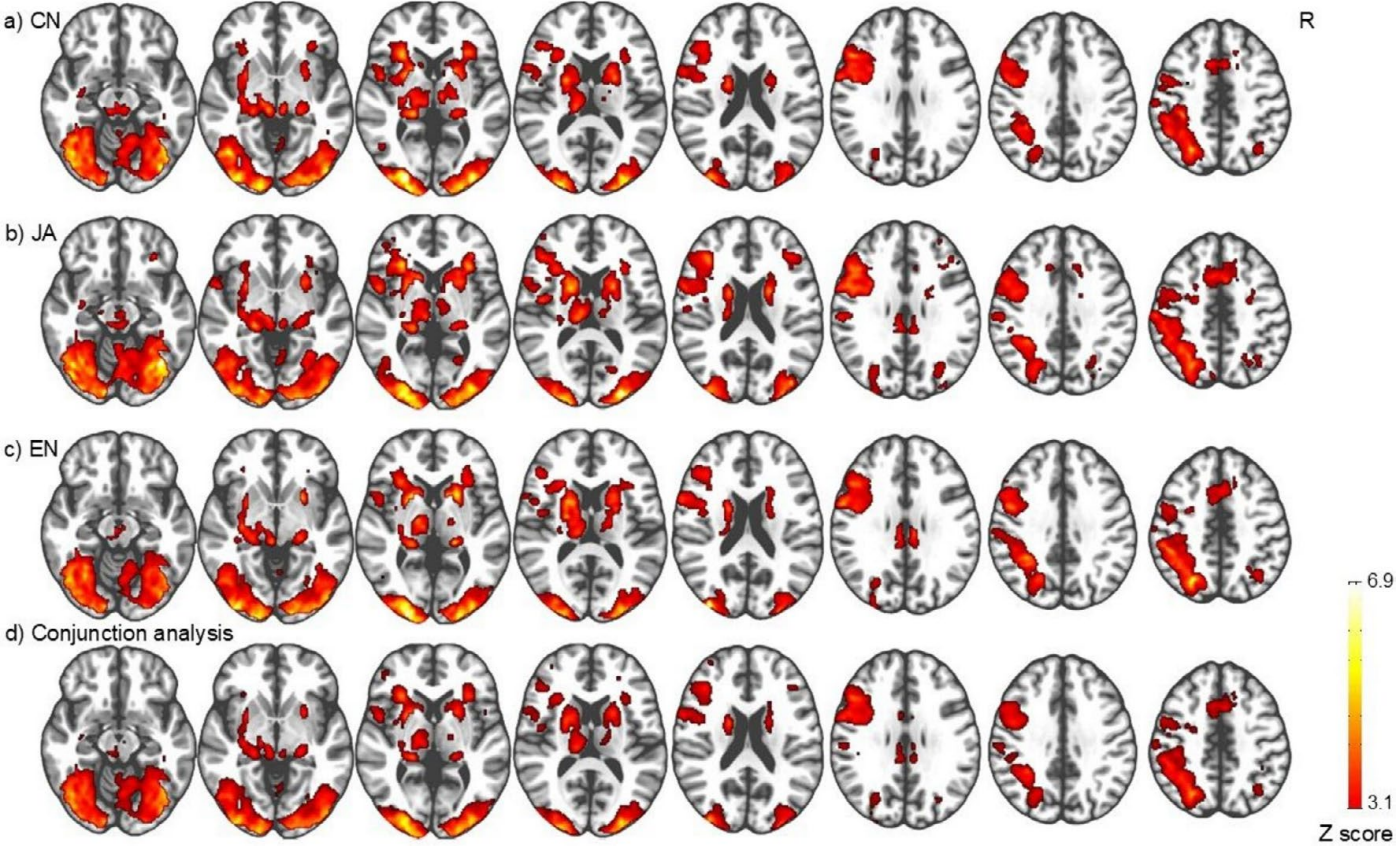
Brain activation for picture naming. Brain activation maps for Chinese (a), Japanese (b), and English (c) and conjunction analysis (d) show overlapping activation across the three languages.

**FIGURE 6:**
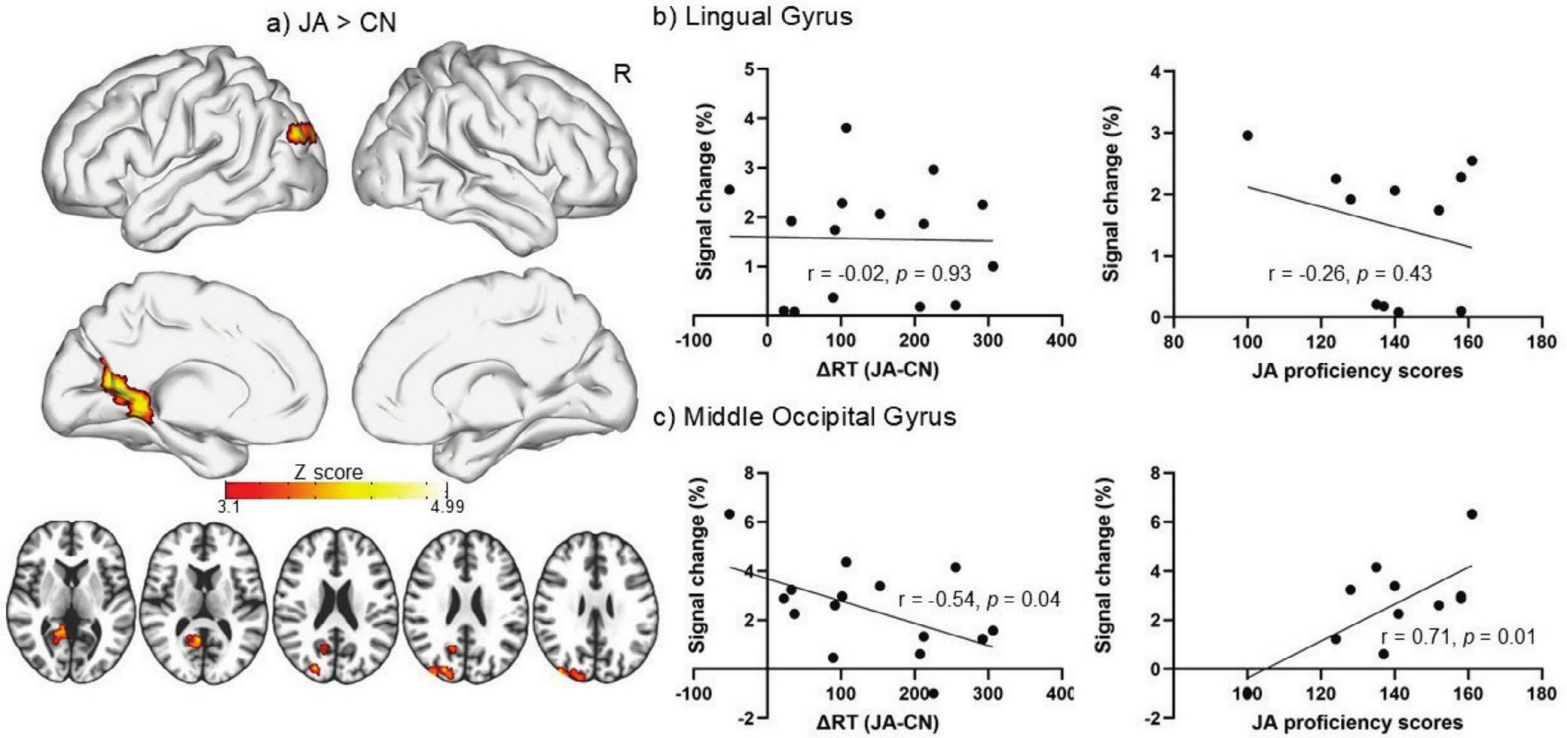
Japanese > Chinese contrast and correlation with behavioral data and Japanese proficiency scores. Greater activation for Japanese language in the left lingual and middle occipital regions (a). LG activity did not reveal a relationship with either ΔRT (JA‐CN) or JA proficiency (b); MOG activity correlated negatively with ΔRT (JA‐CN) and positively with JA proficiency (c).

The three language conditions elicited similar activation patterns in brain regions for lexical retrieval and core language‐related regions, such as the left inferior frontal gyrus, precentral gyrus, superior parietal lobe, occipital lobe, cingulate gyrus, and basal ganglia (Table 1 and Figure 5a–c). The common activation in those regions in the three language conditions was confirmed by a conjunction analysis (Figure 5d). No significant effects of AoA or proficiency were observed at the whole‐brain level for either JA or EN. Direct comparisons between native and non‐native languages showed that JA elicited stronger activation than CN in the left lingual gyrus (LG) and middle occipital gyrus (MOG) (Table 2 and Figure 6a). The MOG activation showed a significant negative correlation with the RT difference between JA and CN (*r* = −0.54, *p* = 0.04) and a positive correlation with JA proficiency (*r* = 0.71, *p* = 0.01) (Figure 6c). LG activity did not reveal a relationship with either the RT difference between JA and CN (*r* = −0.02, *p* = 0.93) or JA proficiency scores (*r* = −0.26, *p* = 0.43) (Figure 6b). No significant differences in brain activation were found in other language pairs.

**TABLE 1:** Brain activation during picture naming task in Chinese, Japanese and English.

Brain regions	MNI coordinates			T‐values	*k* (voxels)
	*x*	*y*	*z*		
Chinese language condition					
Cluster 1					25,667
R middle occipital lobe	29	−95	15	19.35	
L middle occipital lobe	−30	−93	9	17.72	
Cluster 2					20,972
L superior parietal lobe	−27	−63	45	11.98	
L post‐central gyrus	−35	−32	44	11.74	
L inferior frontal gyrus	−30	23	6	12.98	
Cluster 3					2792
L supplementary motor area	−8	6	57	12.66	
L medial frontal gyrus	−12	14	53	11.77	
R superior parietal lobe	32	−62	41	8.94	481
L inferior parietal lobe	−57	−23	27	7.78	158
R inferior parietal lobe	38	−42	44	4.93	66
L middle frontal gyrus	−36	50	14	6.76	85
L anterior cingulate gyrus	−5	5	30	7.51	79
R anterior cingulate gyrus	6	5	29	5.69	39
R caudate head	17	9	5	11.81	2847
Japanese language condition					
Cluster 1					63,556
R middle occipital lobe	38	−90	8	17.33	
L middle occipital lobe	−33	−84	10	16.94	
R cerebellum	39	−72	−26	17.92	
Cluster 2					656
R middle frontal gyrus	33	45	24	5.13	
R inferior frontal gyrus	33	33	5	9.72	
Cluster 3					373
L posterior cingulate gyrus	−6	−30	29	7.1	
R posterior cingulate gyrus	9	−35	29	6.29	
R inferior parietal lobe	32	−45	42	5.02	108
L premotor area	−32	−2	57	6.88	665
L middle frontal gyrus	−33	54	24	4.68	90
R caudate head	20	8	15	12.42	3468
English language condition					
Cluster 1					25,726
R middle occipital lobe	29	−95	15	7.96	
L middle occipital lobe	−27	−89	6	17.34	
Cluster 2					24,499
L superior parietal lobe	−26	−66	45	13.89	
L supplementary motor area	−9	8	57	12.45	
Cluster 3					486
L posterior cingulate gyrus	−6	−20	29	7.21	
R anterior cingulate gyrus	8	−23	30	8.22	
R superior parietal lobe	35	−62	53	7.25	619
R middle frontal gyrus	38	3	60	4.71	124
L inferior frontal gyrus	−42	42	0	4.89	167
R caudate head	17	12	9	11.83	2671
R thalamus	18	−31	3	11.66	419
Conjunction analysis					
Cluster 1					47,101
R middle occipital lobe	29	−95	15	12.67	
L middle occipital lobe	−27	−90	8	14.12	
Cluster 2					235
L posterior cingulate gyrus	−6	−33	27	4.69	
R posterior cingulate gyrus	8	−33	27	4.11	
R superior parietal lobe	33	−62	53	6.87	457
R inferior parietal lobe	36	−42	42	3.74	50
L supplementary motor area	−8	6	57	10.87	3183
R middle frontal gyrus	39	0	57	4.01	143
L inferior frontal gyrus	−30	50	5	9.37	49
R caudate head	21	8	11	7.58	2147
R thalamus	18	−32	2	7.14	364

**TABLE 2:** Brain activation associated Japanese > Chinese contrast.

Brain regions	MNI coordinates			*T*‐values	*k* (voxels)
	*x*	*y*	*z*		
L lingual gyrus	−14	−44	−3	8.24	840
L middle occipital gyrus	−24	−83	23	7.02	414

### 3.3 Representational Similarity Analysis (RSA) fMRI Results

**FIGURE 7:**
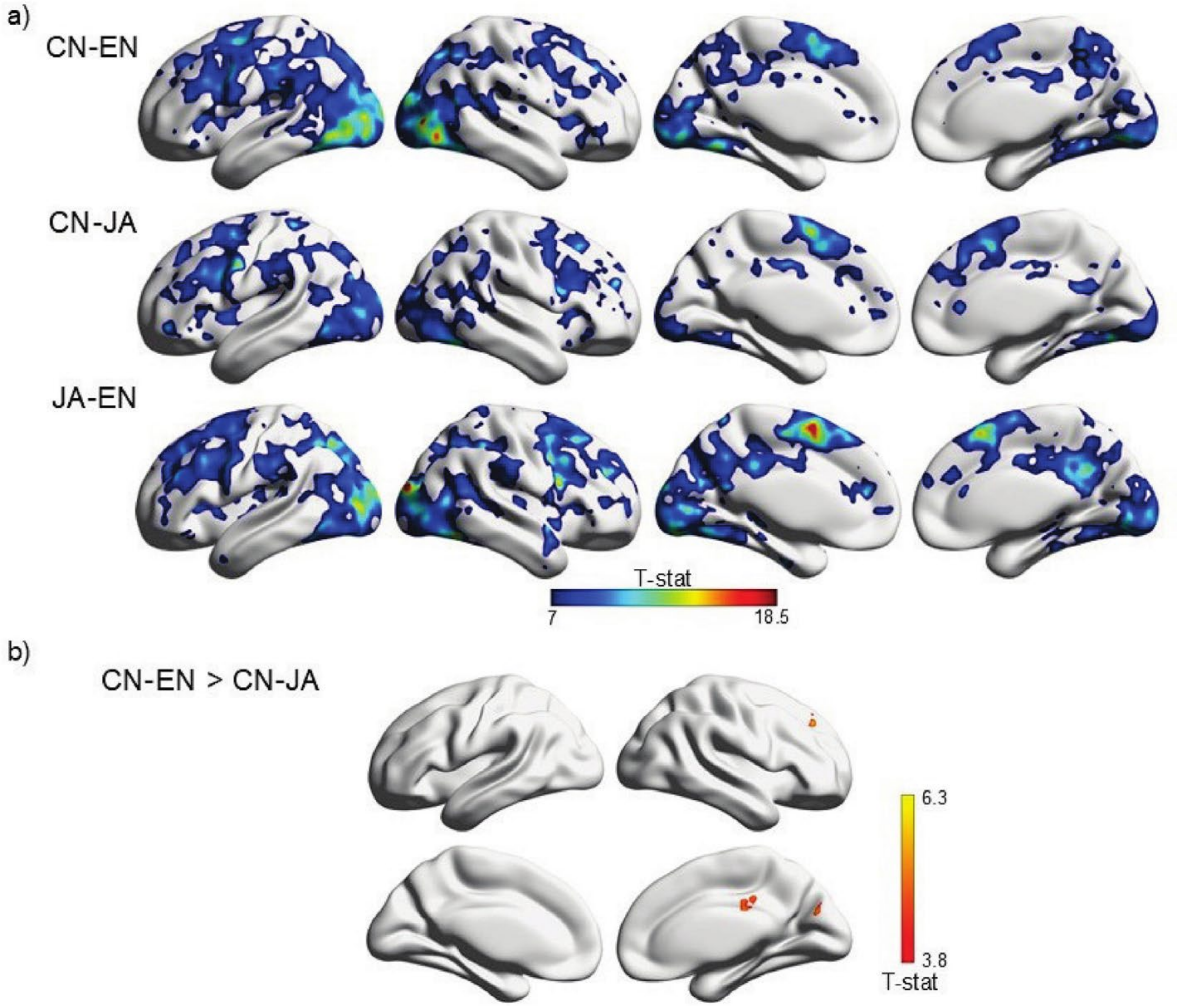
Whole‐brain representational similarity analysis (RSA) (a) Pattern similarity between Chinese–English (CN‐EN), Chinese–Japanese (CN‐JA), and Japanese–English (JA‐EN). (b) Brain regions showing greater pattern similarity between Chinese and English than that of between Chinese and Japanese in the right prefrontal cortex, precuneus and cingulate gyrus.

The whole‐brain searchlight RSA maps revealed widespread cross‐language representational overlap in the bilateral frontal, temporal, and occipital regions. Particularly, the pattern similarity was more extensive and pronounced between CN and EN than between CN and JA (Figure 7a). The greater pattern similarity between CN‐EN than that of CN‐JA was located in the right prefrontal cortex, precuneus, and cingulate gyrus (Table 3, Figure 7b). No significant clusters emerged for either AoA or proficiency for each L2 in the CN‐EN and CN‐JA similarity maps. In addition, because the difference in AoA and proficiency reflected indirectly through RT, given that JA and EN proficiency scores could not be directly comparable, they might contribute to the JA‐EN similarity, we included them as covariates. Again, no significant clusters were observed for either covariate.

**TABLE 3:** Brain activation associated comparison of Chinese‐English > Chinese‐Japanese RSA patterns.

Brain regions	MNI coordinates			*T*‐values	*k* (voxels)
	*x*	*y*	*z*		
R dorsal prefrontal cortex	20	38	38	6.30	74
R precuneus	5	−75	29	5.77	64
Cingulate gyrus	6	−27	32	5.42	71

**FIGURE 8:**
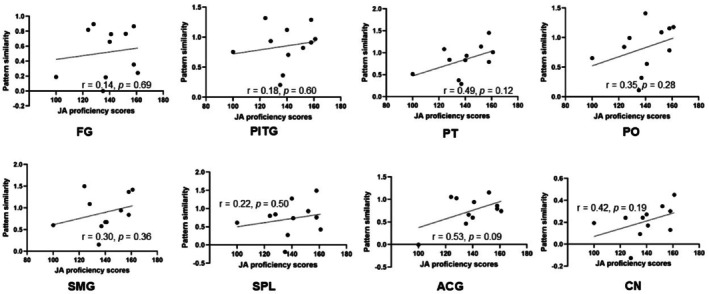
Correlation between ROI‐based RSA of Chinese–Japanese conditions and Japanese proficiency ACG: Anterior cingulate gyrus; CN: Caudate nucleus; FG: Fusiform gyrus; PITG: Posterior inferior temporal gyrus; PO: Pars opercularis; PT: Pars triangularis; SMG: Supra marginal gyrus; SPL: Superior parietal lobe. Trends toward positive correlations that did not reach statistically significant were observed in pars triangularis (*r* = 0.49, *p* = 0.12) and anterior cingulate gyrus (*r* = 0.53, *p* = 0.09).

For the VOI‐based RSA, none of the predefined VOIs showed significant differences in cross‐language pattern similarity among the three pairwise comparisons (all *p* > 0.1) (data not shown). For the VOI‐based CN‐EN pattern similarity, no significant correlation with EN proficiency was found in the eight VOIs (Figure S1). In contrast, for the VOI‐based CN‐JA pattern similarity, positive correlations that did not reach statistical significance were observed in several VOIs, including the pars triangularis (*r* = 0.49, *p* = 0.12) and anterior cingulate gyrus (*r* = 0.53, *p* = 0.09) (Figure 8). Neither the AoA of JA nor that of EN showed any significant correlation with VOI‐based similarity patterns (data not shown).

Additional partial correlation analyses yielded results nearly identical to those of the Pearson correlations. All significant and trend‐level associations remained essentially unchanged and no additional associations reached statistical significance. The complete results are presented in Supplementary Table S6.

### 3.4 Leave‐One‐Out (LOO) Sensitivity Analysis Results

The LOO sensitivity analyses indicated that the primary findings remained stable across all 15 iterations. For the univariate analysis, the peak coordinates of the left occipital activation in each language condition shifted by only a few millimeters, and the *t*‐values remained within a narrow range around the full‐dataset estimates (Table S1). The subtraction of JA to CN contrast in the left LG and MOG showed comparable stability in peak location and cluster extent across iterations (Table S2). For RSA analysis, the whole‐brain searchlight peak coordinates and Fisher's *z*‐scores varied minimally across iterations (Table S3), and the Fisher's *z*‐scores of the VOI‐based RSA showed small variation, with standard deviations between 0.010 and 0.034 (Table S4).

## 4 Discussion

Here, we examined neural representations of L1 Chinese, L2 Japanese, and L2 English during a one‐back covert picture‐naming task to investigate how the multilingual brain organizes distinct languages. Following the recent trend in applying multivariate approaches to multilingual neurocognition, we also conducted RSA for our data to examine representational organization across the three language conditions. Using the same one‐back covert picture‐naming task across the three language conditions, we demonstrated that behavioral efficiency, neural activation, and representational similarity are differentially modulated across non‐native languages. Crucially, L2 JA, which shares logographic characteristics with L1 CN, showed greater interference effects, reflected in reduced behavioral efficiency and altered neural recruitment. Although we could not fully disentangle the effects of linguistic distance from AoA‐ or experience‐related influences, our findings indicated that multilingual brains flexibly adapted assimilation and accommodation mechanisms to orthographic and phonological constraints, thereby advancing mechanistic accounts of cross‐language processing across writing systems.

Behavioral results showed that L2 JA picture naming was associated with longer RT and lower accuracy compared to both L1 CN and L2 EN. Because accuracy in the JA condition was substantially lower, the IES was not considered a reliable composite index. Thus, we cannot fully address the speed‐accuracy trade‐off, and performance was evaluated separately in terms of response time and accuracy. However, the difference in accuracy between JA and EN conditions did not reach statistical significance. Moreover, the RT and accuracy in both L2 conditions were not significantly correlated with L2 proficiency scores. Because the stimuli used in the present study are highly familiar and participants have achieved a high level of L2 proficiency, their task performance may reach a ceiling, limiting the detection of variance across individuals. Moreover, at high proficiency levels, participants may rely on automated lexical access, similar to native‐language processing, rather than on the deliberate use of their L2 skills, thereby diminishing measurable inter‐individual variance in proficiency. Previous studies have shown that language proficiency strongly influences neural and behavioral responses primarily during the early stages of L2 acquisition (Abutalebi and Green 2016; Yang et al. 2015), whereas at advanced levels, task performance tends to be constrained by lexical properties such as frequency or imageability rather than by overall proficiency itself (Li et al. 2019; Perani and Abutalebi 2005).

Regarding AoA, it is difficult to recruit participants from Asian populations who were first exposed to English after the age of 12, given the early introduction of English education in many Asian countries. Participants in the present study showed a significantly late AoA for JA (13–24 years old), in contrast to a much broader early‐to‐late AoA range for EN (3–12 years old). While AoA of JA did not correlate with behavioral performance, AoA of EN showed a robust positive correlation with RT. This finding likely reflected residual maturational constraints. Early exposure to L2 has been shown to facilitate the development of stable orthography‐phonology mappings and to support faster lexical retrieval (Hernandez and Li 2007; Weber‐Fox and Neville 1996). Although all participants had achieved high proficiency in EN, the persistence of the AoA effect may imply that late learners may retain long‐lasting constraints on the efficiency of lexical access. It is also worth noting that, although the statistical analysis did not reach significance, the trend of faster RT and higher accuracy among more JA‐proficient participants aligns with previous reports showing that extensive language experience facilitates the gradual automatization of L2 lexical access (Li et al. 2019). However, this trend should be considered together with AoA, as the late JA AoA in our samples limits the dissociation of proficiency‐ and AoA‐related effects. Therefore, the observed proficiency‐related improvement likely reflected cumulative language experience in late‐acquired learners, rather than independent AoA effects. The reduced behavioral performance observed for JA likely reflected increased processing demands, potentially related to the structural characteristics of its complex writing system. However, given the uniformly late AoA for JA and the exclusion of JA from the IES analysis, the present findings did not allow a definitive separation of writing‐system–related effects from AoA‐ or experience‐related influences. These considerations motivated a cautious interpretation and underscored the need for future studies with more balanced AoA distributions and language‐specific task designs.

The covert picture‐naming task with all three languages recruited canonical language areas, including the left inferior frontal gyrus, precentral gyrus, superior parietal lobule, occipital lobule, thalamus, and basal ganglia, consistent with previous reports (Dong et al. 2005; Indefrey and Levelt 2004; Price 2012). Although the present task requirement to recode language‐specific phonology into a shared alphabetic‐like format might have contributed to some of these observed activations, extensive overlapping activation across these fronto‐parieto‐occipital networks supported the assimilation hypothesis, suggesting that highly proficient multilinguals processed and controlled multiple languages within a shared neural framework (Chee et al. 1999; Perani et al. 1998). In comparison with CN, EN showed no significant brain activation differences, and no brain regions exhibited AoA or proficiency‐related modulation. This finding suggested that the AoA effect observed in behavioral performance mainly limited the speed of lexical access as a maturational constraint, rather than triggering additional neural recruitment once high proficiency had been reached. Put differently, although AoA continued to influence behavioral efficiency, the neural system for EN might already have converged with that of L1, thereby masking experience‐related variability at the activation level (Li et al. 2019; Perani and Abutalebi 2005). RSA captures informational patterns distributed across voxels, thereby revealing representational distinctions between languages even when their overall activation magnitudes appear comparable (Kriegeskorte et al. 2008; Xue et al. 2013). Importantly, whole‐brain searchlight RSA also revealed greater cross‐language pattern similarity between CN and EN than between CN and JA, suggesting that, despite their orthographic differences, EN may share a more systematic phonological‐orthographic mapping structure with CN. This finding aligned with previous research showing that alphabetic L2s can sometimes be more readily assimilated by logographic L1 speakers due to consistent grapheme‐phoneme correspondences (Nelson et al. 2009). However, this finding differed from previous word‐reading studies reporting greater neural pattern similarity between orthographically similar languages (Dong et al. 2021; Kim et al. 2016). One likely explanation was the difference in experimental paradigms. Whereas both previous studies presented written words and therefore directly engaged orthographic processing, the present study used picture naming tasks, which minimized direct orthographic influences and instead emphasized lexical retrieval and phonological access. Picture naming also engaged additional processes, including lexical selection, phonological encoding, and speech planning, so cross‐language similarity was more likely to reflect concept‐to‐spoken‐form mapping than written input similarity. In addition, Japanese Kanji often have multiple context‐dependent pronunciations, whereas Chinese characters generally have a single dominant pronunciation. This ambiguity may increase phonological processing demands during picture naming and reduce neural similarity between Chinese and Japanese despite their orthographic overlap. The present findings therefore did not contradict the previous word‐reading results but instead suggested that neural similarity across languages depends not only on orthographic characteristics but also on task demands and the stage of language processing being examined.

Even after AoA and proficiency were incorporated as covariates in the analysis, the JA condition still elicited stronger activation in the LG and MOG relative to CN. These regions have been known to support orthographic and lexical processing (Tan et al. 2003; Van de Putte et al. 2017). One possible contributor to this difference was the phonological ambiguity of Japanese Kanji, which contrasts with the typically single phonological value of Chinese characters (Lin et al. 2024). Another possibility related to the dual‐script nature of Japanese: Kanji processing is commonly associated with the ventral orthographic pathway, whereas Kana engages more dorsal phonological pathways (Sakurai et al. 2000; Thuy et al. 2004). Thus, the increased recruitment of LG and MOG, particularly MOG, may indicate higher phonological demands for the picture naming‐Kana phonology association. Therefore, these results may reflect higher cognitive demands associated with script‐dependent processing requirements within Japanese (Katsuse et al. 2025). Given the design of the current study, however, we cannot disentangle whether the observed differences arise primarily from Kanji‐related complexity or from additional processing demands associated with Kana. Future work using script‐specific manipulations will be needed to clarify these contributions. Interestingly, the LG and MOG showed dissociation in their relationships with individual differences. Activation in the LG showed no correlation with either the RT difference between JA and CN (ΔRT (JA‐CN)) or JA proficiency, whereas activation in the MOG exhibited a negative correlation with ΔRT and a positive correlation with JA proficiency. These patterns suggested that the LG supported obligatory visual‐orthographic decoding that does not scale with experience, whereas the MOG reflected an experience‐sensitive mechanism that became more engaged as proficiency increased. Put differently, while both regions are recruited to resolve orthographic‐phonological ambiguity, only the MOG appeared to index the efficiency of lexical to phonological access (Jobard et al. 2003; Lai et al. 2021; Martin et al. 2015). In the RSA, the reduced pattern similarity between CN and JA implied that JA engaged distinct neural codes rather than assimilating to the representational format of L1 CN. This dissociation aligned with the accommodation model (Abutalebi and Green 2016; Perfetti et al. 2007), even though both languages share logographic components. When AoA and proficiency for both L2s were included as covariates, no significant whole‐brain effects emerged, which suggested that orthographic distance exerted the strongest influence on cross‐language representational structure. Although not statistically significant, the positive correlations between JA proficiency and representational similarity observed in executive‐control regions (pars triangularis and anterior cingulate cortex) were directionally consistent with a gradual convergence of neural representations as language experience accumulates, potentially through top‐down control mechanisms (Abutalebi et al. 2013; Kheder and Kaan 2021), but this result did not by itself establish ongoing assimilation.

In summary, our findings demonstrated an assimilation pattern for English, whose alphabetic script differs markedly from Chinese, and an accommodation pattern for Japanese, whose logographic system is visually similar yet structurally more complex. Although script‐dependent processing appeared to be the primary driver of this dissociation, the potential contributions of AoA and proficiency could not be fully ruled out. Notably, unlike previous studies reporting facilitation between orthographically similar languages (Dong et al. 2021; Hamada and Koda 2008; Kim et al. 2016; Pae et al. 2017), our results revealed a cross‐language interference effect between two similar logographic systems. Together, these findings suggested that assimilation and accommodation coexist within multilingual brains and are jointly shaped by orthographic distance and individual language‐learning histories.

## 5 Limitations and Future Directions

Several limitations should be acknowledged. First, although the leave‐one‐out sensitivity analyses supported the robustness of the main effects against the influence of individual participants, the modest sample size (*n* = 15) may limit statistical power for detecting subtle effects and may constrain the generalizability of the present findings to the broader population. In addition, four participants lacked Japanese proficiency scores, so proficiency‐related analyses were based on only 11 participants, which may introduce bias and further reduce sensitivity. Second, the covert naming task minimized articulation artifacts but precluded direct verification of naming accuracy at the single‐trial level. Future studies employing overt‐response paradigms or concurrent EMG monitoring could strengthen the behavioral‐neural correspondence. Additionally, longitudinal training studies examining early‐stage learners could clarify the temporal evolution of assimilation–accommodation dynamics during L2 acquisition. Third, model‐based RSA using visual, semantic, and phonological prediction matrices was not directly examined. Such analyses may be informative, but their interpretation would not be straightforward in the present covert picture naming paradigm because multiple cognitive processes contribute to the observed neural representations. Future studies using paradigms specifically designed to dissociate visual, semantic, and phonological representations may help clarify these representational components. Finally, future work using connectivity‐based RSA or graph‐theoretic resting fMRI analysis could further delineate how distributed networks reorganize with experience across multiple L2s.

## 6 Conclusion

This study provided converging behavioral and neuroimaging evidence that the multilingual brain flexibly balances assimilation and accommodation mechanisms depending on linguistic distance and language experience. While L1 Chinese, L2 English, and L2 Japanese shared a core network for lexical‐semantic processing, language‐specific adaptations emerged in visual‐orthographic and executive‐control regions. Though AoA and proficiency may contribute to multilingual neural organization, they do not sufficiently explain the observed pattern similarity differences. Greater pattern similarity between Chinese and English than that between Chinese and Japanese suggested that language structure drives cross‐language neural organization. The observed trend toward convergence between Chinese and Japanese representations with higher proficiency, although not statistically significant, provided preliminary evidence consistent with the plasticity of the multilingual brain and therefore requires confirmation in larger studies. Collectively, these findings advanced our understanding of how the human brain dynamically adapts to multiple languages, providing a neural basis for designing educational strategies tailored to learners' linguistic backgrounds.

## Author Contributions

D.H.D.T. conceptualization, funding acquisition, formal analysis, investigation, methodology, visualization, writing – original draft. N.O. data curation, resources, software, project administration, writing – review and editing. T.A. investigation, writing – review and editing. T.O. investigation, writing – review and editing. T.H. project administration, supervision, methodology, writing – review and editing.

## Funding

This work was supported by Japan Society for the Promotion of Science, 18K00687, 24K10808. Japan Agency for Medical Research and Development, JP23wm0625001.

## Ethics Statement

My article reports human subjects. Recruitment meets scientific requirements and HBMs expectation of inclusivity.

## Conflicts of Interest

The authors declare no conflicts of interest.

## Supporting information


**Table S1:** Leave‐one‐out (LOO) sensitivity analysis of the representative peak activation in Cluster 1 of Table 1.
**Table S2:** Leave‐one‐out (LOO) sensitivity analysis of Japanese > Chinese contrast.
**Table S3:** Leave‐one‐out (LOO) sensitivity analysis of whole‐brain searchlight RSA.
**Table S4:** Leave‐one‐out (LOO) sensitivity analysis of VOI‐based RSA.
**Table S5:** Cross‐language naming agreement for the full stimulus set used in this study.
**Table S6:** Comparison between Pearson and partial correlation analyses.
**Figure S1:** Correlation between ROI‐based RSA of Chinese–English conditions and English proficiency. No significant correlation was found. ACG: Anterior cingulate gyrus; CN: Caudate nucleus; FG: Fusiform gyrus; PITG: Posterior inferior temporal gyrus; PO: Pars opercularis; PT: Pars triangularis; SMG: Supra marginal gyrus; SPL: Superior parietal lobe.

## Data Availability

The data that support the findings of this study are available on request from the corresponding author. The data are not publicly available due to privacy or ethical restrictions.
