# Supplementary material for: Neural Assimilation and Accommodation in Trilinguals: An fMRI Study at 7 T
Source: Hum Brain Mapp. 2026 Jul 31;47(11):e70620. doi: 10.1002/hbm.70620 (PMC13426019; doi:10.1002/hbm.70620)
Supplement: Supplementary file 1 — Table S1: Leave‐one‐out (LOO) sensitivity analysis of the representative peak activation in Cluster 1 of Table 1. Table S2: Leave‐one‐out (LOO) sensitivity analysis of Japanese > Chinese contrast. Table S3: Leave‐one‐out (LOO) sensitivity analysis of whole‐brain searchlight RSA. Table S4: Leave‐one‐out (LOO) sensitivity analysis of VOI‐based RSA. Table S5: Cross‐language naming agreement for the full stimulus set used in this study. Table S6: Comparison between Pearson and partial correlation analyses. Figure S1: Correlation between ROI‐based RSA of Chinese–English conditions and English proficiency. No significant correlation was found. ACG: Anterior cingulate gyrus; CN: Caudate nucleus; FG: Fusiform gyrus; PITG: Posterior inferior temporal gyrus; PO: Pars opercularis; PT: Pars triangularis; SMG: Supra marginal gyrus; SPL: Superior parietal lobe. [file HBM-47-e70620-s001.docx]

**Supplementary Materials**

Neural Assimilation and Accommodation in Trilinguals- an fMRI study at 7T

Dinh Ha Duy Thuy^1,*^, Naoya Oishi^1,*^, Thai Akasaka^1^, Tomohisa Okada^1,2^, Takashi Hanakawa^1,3^

^1^ Human Brain Research Center, Graduate School of Medicine, Kyoto University, Kyoto, Japan

^2^ Support Unit for Functional Magnetic Resonance Imaging, RIKEN Center for Brain Science, Wako 351-0198, Japan

^3^ Department of Integrated Neuroanatomy and Neuroimaging, Graduate School of Medicine, Kyoto University, Kyoto, Japan

*Corresponding author: Dinh Ha Duy Thuy (e-mail: [dinhthuy@kuhp.kyoto-u.ac.jp](mailto:dinhthuy@kuhp.kyoto-u.ac.jp)) and Naoya Oishi (e-mail: [noishi@kuhp.kyoto-u.ac.jp](mailto:noishi@kuhp.kyoto-u.ac.jp))

This file includes:
Tables S1 to S6

Figure S1

**Table S1. Leave-one-out (LOO) sensitivity analysis of the representative peak activation in Cluster 1 of Table 1**

Note: Full dataset denotes the value computed from all fifteen participants. Columns s01 to s15 show the value obtained when the corresponding participant was omitted.

Peak shift (mm) indicates the Euclidean distance between the peak coordinates from each LOO iteration and the original full-dataset analysis.

| Sample Data | Cluster 1 | | | Peak shift (mm) | T-values | ΔT | k  (voxels) | Δk (voxels) |
| --- | --- | --- | --- | --- | --- | --- | --- | --- |
|  | (L middle occipital lobe) | | |  |  |  |  |  |
| **Leave-one-out (LOO) -omitted participant** | x | y | z |  |  |  |  |  |
| **Chinese language condition** |  |  |  |  |  |  |  |  |
| **Full dataset** | **-30** | **-93** | **9** |  | **17.72** |  | **25667** |  |
| LOO Mean |  |  |  | 3.71 | 18.07 |  | 25037.73 |  |
| LOO SD |  |  |  | 2.74 | 0.84 |  | 1823.91 |  |
| s01 | -29 | -95 | 15 | 6.40 | 18.34 | 0.62 | 24625 | -1042 |
| s02 | -35 | -90 | 12 | 6.56 | 17.93 | 0.21 | 24631 | -1036 |
| s03 | -26 | -89 | 9 | 5.66 | 17.79 | 0.07 | 23648 | -2019 |
| s04 | -26 | -87 | 12 | 7.81 | 19.81 | 2.09 | 25151 | -516 |
| s05 | -30 | -93 | 9 | 0.00 | 17.57 | -0.15 | 26350 | 683 |
| s06 | -33 | -90 | 12 | 5.20 | 17.84 | 0.12 | 23542 | -2125 |
| s07 | -30 | -93 | 9 | 0.00 | 19.26 | 1.54 | 24774 | -893 |
| s08 | -30 | -93 | 9 | 0.00 | 17.53 | -0.19 | 22970 | -2697 |
| s09 | -26 | -89 | 12 | 6.40 | 18.13 | 0.41 | 29750 | 4083 |
| s010 | -30 | -90 | 12 | 4.24 | 19.42 | 1.7 | 26806 | 1139 |
| s011 | -30 | -93 | 9 | 0.00 | 16.72 | -1 | 26021 | 354 |
| s012 | -30 | -95 | 11 | 2.83 | 18.03 | 0.31 | 26139 | 472 |
| s013 | -30 | -95 | 9 | 2.00 | 17.65 | -0.07 | 22611 | -3056 |
| s014 | -27 | -90 | 9 | 4.24 | 17.74 | 0.02 | 25117 | -550 |
| s015 | -33 | -90 | 9 | 4.24 | 17.27 | -0.45 | 23431 | -2236 |
| **Japanese language condition** |  |  |  |  |  |  |  |  |
| **Full dataset** | **-33** | **-84** | **10** |  | **16.94** |  | **63556** |  |
| LOO Mean |  |  |  | 6.18 | 16.98 |  | 60413.73 |  |
| LOO SD |  |  |  | 3.54 | 0.91 |  | 2811.82 |  |
| s01 | -38 | -92 | 11 | 9.49 | 16.82 | -0.12 | 61018 | -2538 |
| s02 | -33 | -84 | 14 | 4.00 | 17.52 | 0.58 | 58459 | -5097 |
| s03 | -38 | -88 | 11 | 6.48 | 16.46 | -0.48 | 59732 | -3824 |
| s04 | -33 | -84 | 9 | 1.00 | 16.26 | -0.68 | 59433 | -4123 |
| s05 | -33 | -84 | 15 | 5.00 | 17.08 | 0.14 | 63664 | 108 |
| s06 | -28 | -92 | 12 | 9.64 | 16.74 | -0.2 | 58225 | -5331 |
| s07 | -26 | -92 | 12 | 10.82 | 16.32 | -0.62 | 67337 | 3781 |
| s08 | -33 | -84 | 15 | 5.00 | 15.99 | -0.95 | 57353 | -6203 |
| s09 | -33 | -84 | 11 | 1.00 | 19.18 | 2.24 | 59774 | -3782 |
| s010 | -26 | -92 | 12 | 10.82 | 16.65 | -0.29 | 58225 | -5331 |
| s011 | -33 | -84 | 11 | 1.00 | 18.5 | 1.56 | 59808 | -3748 |
| s012 | -38 | -88 | 11 | 6.48 | 16.87 | -0.07 | 57788 | -5768 |
| s013 | -39 | -82 | 17 | 9.43 | 16.76 | -0.18 | 64841 | 1285 |
| s014 | -30 | -92 | 9 | 8.60 | 17.69 | 0.75 | 60675 | -2881 |
| s015 | -33 | -84 | 14 | 4.00 | 15.9 | -1.04 | 59874 | -3682 |
| **English language condition** |  |  |  |  |  |  |  |  |
| **Full dataset** | **-27** | **-89** | **6** |  | **17.34** |  | **25726** |  |
| LOO Mean |  |  |  | 4.93 | 17.80 |  | 24115.47 |  |
| LOO SD |  |  |  | 4.67 | 1.72 |  | 3314.18 |  |
| s01 | -28 | -92 | 2 | 5.10 | 16.92 | -0.42 | 24968 | -758 |
| s02 | -27 | -89 | 8 | 2.00 | 17.57 | 0.23 | 25376 | -350 |
| s03 | -28 | -92 | 3 | 4.36 | 17.99 | 0.65 | 25192 | -534 |
| s04 | -17 | -86 | 13 | 12.57 | 18.64 | 1.3 | 26033 | 307 |
| s05 | -23 | -98 | 2 | 10.63 | 17.64 | 0.3 | 24641 | -1085 |
| s06 | -25 | -96 | 8 | 7.55 | 17.6 | 0.26 | 23301 | -2425 |
| s07 | -23 | -99 | 8 | 10.95 | 18.9 | 1.56 | 33200 | 7474 |
| s08 | -27 | -89 | 8 | 2.00 | 17.73 | 0.39 | 21747 | -3979 |
| s09 | -27 | -89 | 6 | 0.00 | 23.33 | 5.99 | 20491 | -5235 |
| s010 | -27 | -89 | 6 | 0.00 | 16.93 | -0.41 | 25243 | -483 |
| s011 | -21 | -99 | 8 | 11.83 | 16.72 | -0.62 | 26055 | 329 |
| s012 | -23 | -87 | 8 | 4.90 | 16.15 | -1.19 | 22345 | -3381 |
| s013 | -27 | -89 | 6 | 0.00 | 16.98 | -0.36 | 23078 | -2648 |
| s014 | -27 | -89 | 8 | 2.00 | 17.73 | 0.39 | 20979 | -4747 |
| s015 | -27 | -89 | 6 | 0.00 | 16.21 | -1.13 | 19083 | -6643 |

**Table S2. Leave-one-out (LOO) sensitivity analysis of Japanese > Chinese contrast**

Note: Full dataset denotes the value computed from all fifteen participants. Columns s01 to s15 show the value obtained when the corresponding participant was omitted.

Peak shift (mm) indicates Euclidean distance between the peak coordinates from each LOO iteration and the original full-dataset analysis.

| Sample Data | MNI coordinates (mm) | | | Peak shift (mm) | T-values | ΔT | k  (voxels) | Δk (voxels) |
| --- | --- | --- | --- | --- | --- | --- | --- | --- |
| **Leave-one-out (LOO) –**  **omitted participant** | x | y | z |  |  |  |  |  |
| **L lingual gyrus** |  |  |  |  |  |  |  |  |
| **Full dataset** | **-14** | **-44** | **-3** |  | **8.24** |  | **840** |  |
| LOO Mean |  |  |  | 0.82 | 8.27 |  | 799.47 |  |
| LOO SD |  |  |  | 0.89 | 0.63 |  | 207.41 |  |
| s01 | -14 | -44 | -2 | 1 | 8.48 | 0.24 | 954 | 114 |
| s02 | -14 | -44 | -3 | 0 | 8.05 | -0.19 | 622 | -218 |
| s03 | -15 | -44 | -3 | 1 | 8.62 | 0.38 | 604 | -236 |
| s04 | -14 | -42 | -2 | 2.24 | 8.16 | -0.08 | 703 | -137 |
| s05 | -14 | -44 | -3 | 0 | 7.8 | -0.44 | 1097 | 257 |
| s06 | -14 | -44 | -2 | 1 | 7.92 | -0.32 | 672 | -168 |
| s07 | -14 | -44 | -3 | 0 | 7.67 | -0.57 | 948 | 108 |
| s08 | -14 | -44 | -2 | 1 | 8.09 | -0.15 | 626 | -214 |
| s09 | -14 | -44 | -3 | 0 | 8.47 | 0.23 | 665 | -175 |
| s010 | -12 | -42 | -2 | 3 | 8.37 | 0.13 | 1120 | 280 |
| s011 | -15 | -44 | -3 | 1 | 10.31 | 2.07 | 889 | 49 |
| s012 | -14 | -44 | -3 | 0 | 7.76 | -0.48 | 601 | -239 |
| s013 | -15 | -44 | -3 | 1 | 8.41 | 0.17 | 1168 | 328 |
| s014 | -14 | -44 | -3 | 0 | 8.04 | -0.2 | 648 | -192 |
| s015 | -14 | -44 | -2 | 1 | 7.94 | -0.3 | 675 | -165 |
| **L middle occipital gyrus** |  |  |  |  |  |  |  |  |
| **Full dataset** | **-24** | **-83** | **23** |  | **7.02** |  | **414** |  |
| LOO Mean |  |  |  | 0.89 | 6.98 |  | 383.53 |  |
| LOO SD |  |  |  | 0.77 | 0.43 |  | 78.23 |  |
| s01 | -24 | -83 | 23 | 0.00 | 7.03 | 0.01 | 384 | -30 |
| s02 | -23 | -83 | 23 | 1.00 | 7.67 | 0.65 | 247 | -167 |
| s03 | -23 | -83 | 23 | 1.00 | 6.71 | -0.31 | 272 | -142 |
| s04 | -24 | -84 | 24 | 1.41 | 6.66 | -0.36 | 308 | -106 |
| s05 | -24 | -83 | 23 | 0.00 | 6.54 | -0.48 | 397 | -17 |
| s06 | -23 | -83 | 23 | 1.00 | 6.75 | -0.27 | 287 | -127 |
| s07 | -23 | -83 | 21 | 2.24 | 7.04 | 0.02 | 404 | -10 |
| s08 | -23 | -83 | 23 | 1.00 | 6.59 | -0.43 | 395 | -19 |
| s09 | -24 | -83 | 23 | 0.00 | 6.51 | -0.51 | 364 | -50 |
| s010 | -23 | -84 | 23 | 1.41 | 6.85 | -0.17 | 483 | 69 |
| s011 | -24 | -83 | 23 | 0.00 | 7.1 | 0.08 | 427 | 13 |
| s012 | -23 | -83 | 23 | 1.00 | 7.08 | 0.06 | 381 | -33 |
| s013 | -24 | -83 | 24 | 1.00 | 8.08 | 1.06 | 493 | 79 |
| s014 | -24 | -83 | 23 | 0.00 | 7.05 | 0.03 | 502 | 88 |
| s015 | -23 | -83 | 21 | 2.24 | 7.06 | 0.04 | 409 | -5 |

**Table S3. Leave-one-out (LOO) sensitivity analysis of whole-brain searchlight RSA**

Note: Full dataset denotes the value computed from all fifteen participants. Columns s01 to s15 show the value obtained when the corresponding participant was omitted.

Peak shift (mm) indicates the Euclidean distance between the peak coordinates from each LOO iteration and the original full-dataset analysis.

CN-JA indicates cross-language pattern similarity between Chinese and Japanese; CN-JA, between Chinese and English; and JA-EN between Japanese and English.

| Sample Data | MNI coordinates | | | Peak shift (mm) | Fisher’s  z-scores | Δz |
| --- | --- | --- | --- | --- | --- | --- |
| **Leave-one-out (LOO) –**  **omitted participant** | x | y | z |  |  |  |
| **CN-JA** |  |  |  |  |  |  |
| **Full dataset** | **-25.5** | **-85.5** | **4.5** |  | **1.525** |  |
| LOO Mean |  |  |  | 2.76 | 1.532 |  |
| LOO SD |  |  |  | 1.90 | 0.034 |  |
| s01 | -27 | -85.5 | 6 | 2.12 | 1.555 | 0.030 |
| s02 | -28.5 | -82.5 | 6 | 4.50 | 1.488 | -0.037 |
| s03 | -22.5 | -88.5 | 4.5 | 4.24 | 1.477 | -0.048 |
| s04 | -25.5 | -85.5 | 6 | 1.50 | 1.530 | 0.005 |
| s05 | -25.5 | -85.5 | 6 | 1.50 | 1.561 | 0.035 |
| s06 | -25.5 | -85.5 | 4.5 | 0.00 | 1.536 | 0.011 |
| s07 | -27 | -85.5 | 6 | 2.12 | 1.578 | 0.053 |
| s08 | -31.5 | -84 | 1.5 | 6.87 | 1.546 | 0.021 |
| s09 | -22.5 | -88.5 | 4.5 | 4.24 | 1.587 | 0.062 |
| s10 | -27 | -85.5 | 6 | 2.12 | 1.555 | 0.030 |
| s11 | -25.5 | -85.5 | 4.5 | 0.00 | 1.509 | -0.017 |
| s12 | -22.5 | -88.5 | 4.5 | 4.24 | 1.529 | 0.004 |
| s13 | -27 | -85.5 | 6 | 2.12 | 1.544 | 0.019 |
| s14 | -22.5 | -88.5 | 4.5 | 4.24 | 1.510 | -0.015 |
| s15 | -25.5 | -85.5 | 6 | 1.50 | 1.477 | -0.048 |
| **CN-EN** |  |  |  |  |  |  |
| **Full dataset** | **-21** | **-91.5** | **7.5** |  | **1.605** |  |
| LOO Mean |  |  |  | 0.33 | 1.606 |  |
| LOO SD |  |  |  | 1.28 | 0.033 |  |
| s01 | -21 | -91.5 | 7.5 | 0.00 | 1.636 | 0.031 |
| s02 | -21 | -91.5 | 7.5 | 0.00 | 1.604 | -0.001 |
| s03 | -21 | -91.5 | 7.5 | 0.00 | 1.578 | -0.028 |
| s04 | -21 | -91.5 | 7.5 | 0.00 | 1.616 | 0.010 |
| s05 | -21 | -91.5 | 7.5 | 0.00 | 1.637 | 0.031 |
| s06 | -21 | -91.5 | 7.5 | 0.00 | 1.565 | -0.040 |
| s07 | -25.5 | -90 | 9 | 4.97 | 1.635 | 0.030 |
| s08 | -21 | -91.5 | 7.5 | 0.00 | 1.642 | 0.036 |
| s09 | -21 | -91.5 | 7.5 | 0.00 | 1.641 | 0.035 |
| s10 | -21 | -91.5 | 7.5 | 0.00 | 1.623 | 0.018 |
| s11 | -21 | -91.5 | 7.5 | 0.00 | 1.555 | -0.050 |
| s12 | -21 | -91.5 | 7.5 | 0.00 | 1.576 | -0.029 |
| s13 | -21 | -91.5 | 7.5 | 0.00 | 1.625 | 0.019 |
| s14 | -21 | -91.5 | 7.5 | 0.00 | 1.601 | -0.004 |
| s15 | -21 | -91.5 | 7.5 | 0.00 | 1.551 | -0.055 |
| **JA-EN** |  |  |  |  |  |  |
| **Full dataset** | **-24** | **-87** | **4.5** |  | **1.647** |  |
| LOO Mean |  |  |  | 0.99 | 1.651 |  |
| LOO SD |  |  |  | 1.10 | 0.034 |  |
| s01 | -24 | -87 | 4.5 | 0.00 | 1.685 | 0.039 |
| s02 | -24 | -87 | 4.5 | 0.00 | 1.636 | -0.011 |
| s03 | -22.5 | -88.5 | 4.5 | 2.12 | 1.616 | -0.030 |
| s04 | -22.5 | -88.5 | 4.5 | 2.12 | 1.604 | -0.042 |
| s05 | -24 | -87 | 4.5 | 0.00 | 1.648 | 0.002 |
| s06 | -24 | -87 | 4.5 | 0.00 | 1.657 | 0.011 |
| s07 | -24 | -87 | 4.5 | 0.00 | 1.685 | 0.039 |
| s08 | -22.5 | -88.5 | 4.5 | 2.12 | 1.670 | 0.024 |
| s09 | -22.5 | -88.5 | 4.5 | 2.12 | 1.732 | 0.085 |
| s10 | -24 | -87 | 4.5 | 0.00 | 1.623 | -0.024 |
| s11 | -24 | -87 | 4.5 | 0.00 | 1.631 | -0.015 |
| s12 | -22.5 | -88.5 | 4.5 | 2.12 | 1.669 | 0.022 |
| s13 | -25.5 | -85.5 | 4.5 | 2.12 | 1.645 | -0.001 |
| s14 | -24 | -87 | 4.5 | 0.00 | 1.658 | 0.012 |
| s15 | -22.5 | -88.5 | 4.5 | 2.12 | 1.606 | -0.040 |

**Table S4. Leave-one-out (LOO) sensitivity analysis of VOI-based RSA**

Note: Full dataset denotes the value computed from all fifteen participants. Columns s01 to s15 show the value obtained when the corresponding participant was omitted.

All values are Fisher’s z-scores.

FG: fusiform gyrus; PITG: posterior inferior temporal gyrus; PT: pars triangularis; PO: pars opercularis; SPL: superior parietal lobe; SMG: supra marginal gyrus; ACG: anterior cingulate gyrus; CN: caudate nucleus.

| **Pair** | **VOI** | **Full dataset** | **Leave-one-out (LOO) -omitted participant** | | | | | | | | | | | | | | | | | | |
| --- | --- | --- | --- | --- | --- | --- | --- | --- | --- | --- | --- | --- | --- | --- | --- | --- | --- | --- | --- | --- | --- |
|  |  |  | LOO Mean | LOO SD | Min | Max | s01 | s02 | s03 | s04 | s05 | s06 | s07 | s08 | s09 | s10 | s11 | s12 | s13 | s14 | s15 |
| **CN_EN** | FG | 0.572 | 0.572 | 0.025 | 0.541 | 0.616 | 0.541 | 0.558 | 0.562 | 0.599 | 0.595 | 0.549 | 0.589 | 0.542 | 0.592 | 0.616 | 0.584 | 0.548 | 0.581 | 0.587 | 0.542 |
|  | PITG | 0.841 | 0.841 | 0.023 | 0.800 | 0.873 | 0.844 | 0.840 | 0.833 | 0.844 | 0.873 | 0.809 | 0.855 | 0.835 | 0.869 | 0.864 | 0.846 | 0.828 | 0.800 | 0.869 | 0.807 |
|  | PT | 0.730 | 0.730 | 0.034 | 0.684 | 0.779 | 0.713 | 0.713 | 0.748 | 0.714 | 0.777 | 0.703 | 0.768 | 0.693 | 0.759 | 0.771 | 0.684 | 0.713 | 0.724 | 0.779 | 0.692 |
|  | PO | 0.726 | 0.726 | 0.025 | 0.693 | 0.768 | 0.705 | 0.724 | 0.729 | 0.693 | 0.749 | 0.720 | 0.768 | 0.694 | 0.752 | 0.748 | 0.700 | 0.707 | 0.710 | 0.764 | 0.718 |
|  | SMG | 0.839 | 0.839 | 0.027 | 0.795 | 0.878 | 0.849 | 0.816 | 0.841 | 0.807 | 0.872 | 0.795 | 0.865 | 0.849 | 0.869 | 0.866 | 0.814 | 0.822 | 0.828 | 0.878 | 0.819 |
|  | SPL | 0.812 | 0.812 | 0.029 | 0.763 | 0.868 | 0.808 | 0.810 | 0.784 | 0.836 | 0.839 | 0.789 | 0.855 | 0.788 | 0.837 | 0.868 | 0.763 | 0.810 | 0.782 | 0.810 | 0.808 |
|  | ACG | 0.665 | 0.665 | 0.027 | 0.613 | 0.703 | 0.678 | 0.647 | 0.679 | 0.680 | 0.688 | 0.641 | 0.681 | 0.613 | 0.703 | 0.684 | 0.644 | 0.643 | 0.661 | 0.703 | 0.629 |
|  | CN | 0.233 | 0.233 | 0.012 | 0.215 | 0.251 | 0.231 | 0.219 | 0.248 | 0.227 | 0.249 | 0.230 | 0.235 | 0.228 | 0.237 | 0.251 | 0.215 | 0.247 | 0.227 | 0.235 | 0.216 |
| **CN_JA** | FG | 0.471 | 0.471 | 0.024 | 0.440 | 0.505 | 0.451 | 0.440 | 0.458 | 0.487 | 0.491 | 0.443 | 0.492 | 0.450 | 0.489 | 0.505 | 0.480 | 0.441 | 0.491 | 0.503 | 0.446 |
|  | PITG | 0.769 | 0.769 | 0.024 | 0.730 | 0.810 | 0.774 | 0.770 | 0.744 | 0.755 | 0.804 | 0.732 | 0.798 | 0.766 | 0.789 | 0.810 | 0.759 | 0.757 | 0.770 | 0.781 | 0.730 |
|  | PT | 0.739 | 0.739 | 0.028 | 0.688 | 0.786 | 0.726 | 0.718 | 0.733 | 0.720 | 0.786 | 0.736 | 0.771 | 0.711 | 0.754 | 0.765 | 0.688 | 0.732 | 0.755 | 0.779 | 0.715 |
|  | PO | 0.728 | 0.728 | 0.028 | 0.679 | 0.772 | 0.740 | 0.723 | 0.679 | 0.695 | 0.755 | 0.724 | 0.757 | 0.702 | 0.748 | 0.772 | 0.697 | 0.709 | 0.733 | 0.762 | 0.719 |
|  | SMG | 0.823 | 0.823 | 0.031 | 0.774 | 0.870 | 0.833 | 0.785 | 0.833 | 0.780 | 0.859 | 0.821 | 0.840 | 0.814 | 0.842 | 0.870 | 0.784 | 0.804 | 0.838 | 0.860 | 0.774 |
|  | SPL | 0.695 | 0.695 | 0.033 | 0.638 | 0.758 | 0.692 | 0.644 | 0.653 | 0.714 | 0.724 | 0.690 | 0.725 | 0.678 | 0.729 | 0.758 | 0.638 | 0.684 | 0.700 | 0.703 | 0.687 |
|  | ACG | 0.626 | 0.626 | 0.026 | 0.588 | 0.671 | 0.603 | 0.626 | 0.628 | 0.618 | 0.655 | 0.614 | 0.623 | 0.588 | 0.666 | 0.637 | 0.609 | 0.597 | 0.671 | 0.656 | 0.595 |
|  | CN | 0.172 | 0.172 | 0.012 | 0.152 | 0.193 | 0.172 | 0.162 | 0.165 | 0.152 | 0.181 | 0.175 | 0.167 | 0.159 | 0.193 | 0.177 | 0.162 | 0.193 | 0.170 | 0.181 | 0.167 |
| **JA_EN** | FG | 0.550 | 0.550 | 0.020 | 0.519 | 0.574 | 0.546 | 0.524 | 0.553 | 0.572 | 0.563 | 0.519 | 0.571 | 0.523 | 0.567 | 0.574 | 0.559 | 0.523 | 0.568 | 0.561 | 0.533 |
|  | PITG | 0.770 | 0.770 | 0.021 | 0.737 | 0.805 | 0.780 | 0.781 | 0.758 | 0.759 | 0.805 | 0.744 | 0.798 | 0.737 | 0.786 | 0.790 | 0.758 | 0.768 | 0.771 | 0.784 | 0.738 |
|  | PT | 0.766 | 0.766 | 0.033 | 0.695 | 0.813 | 0.770 | 0.775 | 0.776 | 0.730 | 0.798 | 0.765 | 0.813 | 0.716 | 0.763 | 0.803 | 0.695 | 0.766 | 0.769 | 0.804 | 0.740 |
|  | PO | 0.641 | 0.641 | 0.029 | 0.592 | 0.697 | 0.648 | 0.653 | 0.630 | 0.604 | 0.649 | 0.632 | 0.679 | 0.592 | 0.639 | 0.697 | 0.603 | 0.638 | 0.636 | 0.682 | 0.634 |
|  | SMG | 0.801 | 0.801 | 0.029 | 0.742 | 0.857 | 0.823 | 0.783 | 0.813 | 0.742 | 0.830 | 0.796 | 0.812 | 0.775 | 0.812 | 0.815 | 0.774 | 0.783 | 0.820 | 0.857 | 0.776 |
|  | SPL | 0.838 | 0.838 | 0.028 | 0.781 | 0.874 | 0.866 | 0.828 | 0.802 | 0.874 | 0.861 | 0.824 | 0.829 | 0.781 | 0.860 | 0.866 | 0.803 | 0.823 | 0.842 | 0.854 | 0.853 |
|  | ACG | 0.684 | 0.684 | 0.032 | 0.624 | 0.728 | 0.696 | 0.681 | 0.712 | 0.708 | 0.697 | 0.680 | 0.691 | 0.624 | 0.712 | 0.689 | 0.633 | 0.644 | 0.714 | 0.728 | 0.650 |
|  | CN | 0.253 | 0.253 | 0.010 | 0.239 | 0.273 | 0.248 | 0.252 | 0.273 | 0.262 | 0.262 | 0.256 | 0.244 | 0.239 | 0.259 | 0.263 | 0.243 | 0.254 | 0.246 | 0.256 | 0.240 |

**Table S5.** **Cross-language naming agreement for the full stimulus set used in this study**

Naming agreement was assessed in a separate behavioral experiment in an independent sample of ten native Chinese speakers who were highly proficient in Japanese and English, none of whom participated in the fMRI experiment. Each participant overtly named all stimuli in Chinese, Japanese and English. Three indices were computed for every item in each language, namely the response percentage, the strict naming agreement, and an entropy-based index of name disagreement (H).
Response percentage was calculated as the percentage of participants who produced any naming response for a given item, regardless of whether the response was correct. Unlike monolingual picture-naming norms, the present study involved picture naming in both the native language (Chinese) and two second languages (Japanese and English). Because response percentages in Chinese were close to 100% for almost all items and no item was reported as an ‘don’t know object’, failure to produce a response occurred predominantly in the second language tasks and was therefore considered to indicate limited second-language vocabulary rather than disagreement in object naming or failure to recognize the depicted object. Response percentage was accordingly reported separately from strict naming agreement to distinguish lexical accessibility from naming consistency.

Before calculating the strict naming agreement and the H statistic, responses were normalized within each language. Only true synonyms referring to the same concept, for example the Chinese forms 衬衫 (chenshan) and 衬衣 (chenyi) for shirt, were merged into a single category, whereas generic, basic-level, and subordinate-level names, such as fish and goldfish, were retained as separate responses because they differ in lexical specificity and phonological form. Because the present fMRI task required judging whether the initial phoneme of each picture name matched that of the preceding item, strict naming agreement was adopted as the primary measure. It was calculated as the percentage of participants who produced the most common name for that item among those who provided any response. For example, if six participants provided an English name and all six produced iron, the response percentage was 60% and the strict naming agreement was 100%. The H statistic was calculated from the distribution of all naming responses for each item, following Nishimoto et al. (2005).

Across the full stimulus set, the mean strict naming agreement was 89% for Chinese, 87% for Japanese, and 90% for English, indicating high cross-language naming consistency.

Note: Resp. (%) indicates the response percentage; NAst(%) the strict naming agreement; H the statistics of name disagreement

| **Chinese name** | **Resp. (%)** | **NA_st_ (%)** | **H** | **Japanese name  (Katakana)** | **Resp. (%)** | **NA_st_ (%)** | **H** | **English name** | **Resp. (%)** | **NA_st_ (%)** | **H** |
| --- | --- | --- | --- | --- | --- | --- | --- | --- | --- | --- | --- |
| 鸭子 | 100 | 100.0 | 0.00 | アヒル | 100 | 100.0 | 0.00 | Duck | 100 | 100.0 | 0.00 |
| 熨斗 | 100 | 100.0 | 0.47 | アイロン | 60 | 100.0 | 0.00 | Iron | 70 | 100.0 | 0.00 |
| 蚂蚁 | 100 | 100.0 | 0.00 | アリ | 90 | 100.0 | 0.00 | Ant | 100 | 100.0 | 0.00 |
| 脚 | 100 | 100.0 | 0.00 | アシ | 90 | 100.0 | 0.00 | Foot | 100 | 100.0 | 0.00 |
| 芦笋 | 90 | 66.7 | 1.43 | アスパラ | 70 | 71.4 | 1.15 | Asparagus | 80 | 50.0 | 1.41 |
| 香蕉 | 100 | 100.0 | 0.00 | バナナ | 100 | 100.0 | 0.00 | Banana | 100 | 100.0 | 0.00 |
| 玫瑰 | 100 | 100.0 | 0.00 | バラ | 100 | 80.0 | 0.72 | Rose | 100 | 100.0 | 0.00 |
| 排球 | 100 | 80.0 | 0.92 | バレーボール | 100 | 90.0 | 0.47 | Ball | 100 | 100.0 | 0.00 |
| 公交车 | 100 | 90.0 | 0.47 | バス | 100 | 100.0 | 0.00 | Bus | 100 | 100.0 | 0.00 |
| 蚱蜢 | 100 | 50.0 | 1.69 | バッタ | 60 | 83.3 | 0.65 | Grasshopper | 50 | 60.0 | 1.37 |
| 棒球棒 | 100 | 90.0 | 0.72 | バット | 80 | 100.0 | 0.00 | Baseball bat | 80 | 75.0 | 0.81 |
| 床 | 100 | 100.0 | 0.00 | ベッド | 100 | 100.0 | 0.00 | Bed | 100 | 100.0 | 0.00 |
| 长椅 | 100 | 70.0 | 0.88 | ベンチ | 100 | 60.0 | 0.97 | Bench | 100 | 100.0 | 0.00 |
| 腰带 | 100 | 80.0 | 0.72 | ベルト | 70 | 100.0 | 0.00 | Belt | 90 | 100.0 | 0.00 |
| 瓶子 | 100 | 70.0 | 0.88 | ビン | 100 | 70.0 | 0.88 | Bottle | 100 | 90.0 | 0.47 |
| 纽扣 | 100 | 100.0 | 0.00 | ボタン | 50 | 100.0 | 0.00 | Button | 70 | 100.0 | 0.00 |
| 圆珠笔 | 100 | 50.0 | 1.00 | ボールペン | 100 | 100.0 | 0.00 | Pen | 100 | 100.0 | 0.00 |
| 帽子 | 100 | 100.0 | 0.00 | ボウシ | 90 | 100.0 | 0.00 | Hat | 100 | 100.0 | 0.00 |
| 葡萄 | 100 | 100.0 | 0.00 | ブドウ | 100 | 90.0 | 0.47 | Grapes | 100 | 100.0 | 0.00 |
| 秋千 | 100 | 50.0 | 0.86 | ブランコ | 10 | 100.0 | 0.00 | Swing | 30 | 100.0 | 0.00 |
| 刷子 | 100 | 100.0 | 0.00 | ブラシ | 90 | 100.0 | 0.00 | Brush | 100 | 100.0 | 0.00 |
| 猪 | 100 | 100.0 | 0.00 | ブタ | 100 | 100.0 | 0.00 | Pig | 100 | 100.0 | 0.00 |
| 蝴蝶 | 100 | 100.0 | 0.00 | チョウ | 100 | 80.0 | 0.72 | Butterfly | 100 | 100.0 | 0.00 |
| 白萝卜 | 100 | 60.0 | 0.97 | ダイコン | 100 | 70.0 | 1.16 | Japanese radish | 80 | 62.5 | 1.30 |
| 壁炉 | 100 | 70.0 | 1.16 | ダンロ | 60 | 66.7 | 1.25 | Fireplace | 70 | 57.1 | 0.99 |
| 灯泡 | 100 | 100.0 | 0.00 | デンキュウ | 70 | 100.0 | 0.00 | Light bulb | 70 | 100.0 | 0.00 |
| 电车 | 100 | 50.0 | 1.36 | デンシャ | 100 | 100.0 | 0.00 | Train | 100 | 100.0 | 0.00 |
| 电话 | 100 | 100.0 | 0.00 | デンワ | 100 | 100.0 | 0.00 | Telephone | 100 | 100.0 | 0.00 |
| 书桌 | 100 | 90.0 | 0.47 | デスク | 100 | 50.0 | 1.49 | Desk | 100 | 70.0 | 0.88 |
| 门 | 100 | 100.0 | 0.00 | ドア | 100 | 90.0 | 0.47 | Door | 100 | 100.0 | 0.00 |
| 连衣裙 | 100 | 80.0 | 0.92 | ドレス | 100 | 70.0 | 0.88 | Dress | 100 | 70.0 | 0.88 |
| 虾 | 100 | 80.0 | 0.92 | エビ | 80 | 100.0 | 0.00 | Shrimp | 90 | 100.0 | 0.00 |
| 铅笔 | 100 | 100.0 | 0.00 | エンピツ | 100 | 90.0 | 0.47 | Pencil | 100 | 100.0 | 0.00 |
| 烟囱 | 100 | 100.0 | 0.00 | エントツ | 60 | 66.7 | 0.92 | Chimney | 30 | 66.7 | 0.92 |
| 叉子 | 100 | 100.0 | 0.00 | フォーク | 100 | 100.0 | 0.00 | Fork | 100 | 100.0 | 0.00 |
| 画笔 | 100 | 80.0 | 0.92 | フデ | 90 | 88.9 | 0.50 | Paintbrush | 80 | 100.0 | 0.00 |
| 哨子 | 100 | 100.0 | 0.00 | フエ | 10 | 100.0 | 0.00 | Whistle | 50 | 100.0 | 0.00 |
| 猫头鹰 | 100 | 100.0 | 0.00 | フクロウ | 60 | 66.7 | 0.92 | Owl | 100 | 100.0 | 0.00 |
| 平底锅 | 100 | 80.0 | 0.72 | フライパン | 100 | 80.0 | 0.92 | Frying pan | 100 | 80.0 | 0.72 |
| 气球 | 100 | 100.0 | 0.00 | フウセン | 100 | 80.0 | 0.72 | Balloon | 100 | 100.0 | 0.00 |
| 信封 | 100 | 100.0 | 0.00 | フウトウ | 100 | 50.0 | 1.00 | Envelope | 100 | 90.0 | 0.47 |
| 风车 | 100 | 100.0 | 0.00 | フウシャ | 20 | 100.0 | 0.00 | Windmill | 30 | 66.7 | 0.92 |
| 吉他 | 100 | 100.0 | 0.00 | ギター | 100 | 100.0 | 0.00 | Guitar | 100 | 100.0 | 0.00 |
| 大猩猩 | 100 | 80.0 | 0.72 | ゴリラ | 100 | 60.0 | 1.37 | Gorilla | 100 | 50.0 | 1.49 |
| 酒杯 | 100 | 80.0 | 0.92 | グラス | 100 | 100.0 | 0.00 | Wineglass | 100 | 90.0 | 0.47 |
| 爱心 | 100 | 100.0 | 0.00 | ハート | 100 | 80.0 | 0.72 | Heart | 100 | 100.0 | 0.00 |
| 牙刷 | 100 | 100.0 | 0.00 | ハブラシ | 100 | 100.0 | 0.00 | Toothbrush | 100 | 100.0 | 0.00 |
| 蜜蜂 | 100 | 90.0 | 0.47 | ハチ | 100 | 70.0 | 0.88 | Bee | 100 | 90.0 | 0.47 |
| 苍蝇 | 100 | 90.0 | 0.47 | ハエ | 70 | 71.4 | 0.75 | Fly | 80 | 100.0 | 0.00 |
| 纸箱 | 100 | 50.0 | 1.00 | ハコ | 100 | 70.0 | 0.88 | Box | 100 | 100.0 | 0.00 |
| 天鹅 | 100 | 70.0 | 0.88 | ハクチョウ | 80 | 75.0 | 0.81 | Swan | 80 | 62.5 | 0.95 |
| 口琴 | 90 | 77.8 | 0.99 | ハーモニカ | 50 | 40.0 | 1.06 | Harmonica | 40 | 50.0 | 1.00 |
| 花 | 100 | 100.0 | 0.00 | ハナ | 100 | 100.0 | 0.00 | Flower | 100 | 100.0 | 0.00 |
| 鼻子 | 100 | 100.0 | 0.00 | ハナ | 100 | 100.0 | 0.00 | Nose | 100 | 100.0 | 0.00 |
| 衣架 | 100 | 90.0 | 0.47 | ハンガー | 60 | 100.0 | 0.00 | Hanger | 60 | 100.0 | 0.00 |
| 手帕 | 100 | 90.0 | 0.47 | ハンカチ | 90 | 100.0 | 0.00 | Handkerchief | 90 | 88.9 | 0.50 |
| 吊床 | 100 | 100.0 | 0.00 | ハンモック | 30 | 100.0 | 0.00 | Hammock | 10 | 100.0 | 0.00 |
| 叶子 | 100 | 80.0 | 0.92 | ハ | 90 | 100.0 | 0.00 | Leaf | 100 | 100.0 | 0.00 |
| 针 | 100 | 100.0 | 0.00 | ハリ | 70 | 100.0 | 0.00 | Needle | 70 | 100.0 | 0.00 |
| 剪刀 | 100 | 100.0 | 0.00 | ハサミ | 100 | 100.0 | 0.00 | Scissors | 90 | 100.0 | 0.00 |
| 梯子 | 100 | 100.0 | 0.00 | ハシゴ | 70 | 71.4 | 0.86 | Ladder | 70 | 100.0 | 0.00 |
| 旗子 | 100 | 100.0 | 0.00 | ハタ | 100 | 70.0 | 0.88 | Flag | 100 | 100.0 | 0.00 |
| 鸽子 | 100 | 90.0 | 0.47 | ハト | 100 | 60.0 | 0.97 | Pigeon | 100 | 50.0 | 1.00 |
| 蛇 | 100 | 90.0 | 0.47 | ヘビ | 100 | 100.0 | 0.00 | Snake | 100 | 100.0 | 0.00 |
| 直升机 | 100 | 100.0 | 0.00 | ヘリコプター | 100 | 100.0 | 0.00 | Helicopter | 100 | 100.0 | 0.00 |
| 飞机 | 100 | 100.0 | 0.00 | ヒコウキ | 100 | 100.0 | 0.00 | Airplane | 100 | 100.0 | 0.00 |
| 向日葵 | 100 | 100.0 | 0.00 | ヒマワリ | 100 | 80.0 | 0.72 | Sunflower | 90 | 77.8 | 0.76 |
| 食指 | 100 | 60.0 | 0.97 | ヒトサシユビ | 100 | 100.0 | 0.00 | Finger | 100 | 100.0 | 0.00 |
| 书 | 100 | 100.0 | 0.00 | ホン | 100 | 100.0 | 0.00 | Book | 100 | 100.0 | 0.00 |
| 书架 | 100 | 100.0 | 0.00 | ホンダナ | 100 | 100.0 | 0.00 | Bookshelf | 100 | 100.0 | 0.00 |
| 骨头 | 100 | 100.0 | 0.00 | ホネ | 100 | 100.0 | 0.00 | Bone | 100 | 100.0 | 0.00 |
| 星星 | 100 | 60.0 | 0.97 | ホシ | 100 | 100.0 | 0.00 | Star | 100 | 100.0 | 0.00 |
| 菜刀 | 100 | 80.0 | 0.52 | ホウチョウ | 100 | 70.0 | 0.88 | Kitchen knife | 100 | 100.0 | 0.00 |
| 扫把 | 100 | 90.0 | 0.47 | ホウキ | 70 | 100.0 | 0.00 | Broom | 50 | 60.0 | 0.97 |
| 草莓 | 100 | 100.0 | 0.00 | イチゴ | 100 | 100.0 | 0.00 | Strawberry | 100 | 100.0 | 0.00 |
| 水井 | 100 | 70.0 | 0.88 | イド | 70 | 100.0 | 0.00 | Well | 30 | 100.0 | 0.00 |
| 房子 | 100 | 100.0 | 0.00 | イエ | 100 | 70.0 | 0.88 | House | 100 | 100.0 | 0.00 |
| 锚 | 100 | 90.0 | 0.47 | イカリ | 30 | 100.0 | 0.00 | Anchor | 30 | 66.7 | 0.92 |
| 狗 | 100 | 100.0 | 0.00 | イヌ | 100 | 100.0 | 0.00 | Dog | 100 | 100.0 | 0.00 |
| 海豚 | 100 | 100.0 | 0.00 | イルカ | 90 | 66.7 | 0.74 | Dolphin | 90 | 100.0 | 0.00 |
| 椅子 | 100 | 90.0 | 0.47 | イス | 100 | 100.0 | 0.00 | Chair | 100 | 100.0 | 0.00 |
| 土豆 | 100 | 90.0 | 0.47 | ジャガイモ | 100 | 100.0 | 0.00 | Potato | 100 | 100.0 | 0.00 |
| 水龙头 | 100 | 100.0 | 0.00 | ジャグチ | 70 | 100.0 | 0.00 | Faucet | 40 | 75.0 | 0.81 |
| 自行车 | 100 | 100.0 | 0.00 | ジテンシャ | 100 | 100.0 | 0.00 | Bicycle | 100 | 100.0 | 0.00 |
| 洒水壶 | 100 | 50.0 | 1.49 | ジョウロ | 10 | 100.0 | 0.00 | Watering can | 10 | 100.0 | 0.00 |
| 蚊子 | 100 | 70.0 | 0.80 | カ | 70 | 85.7 | 0.59 | Mosquito | 80 | 87.5 | 0.54 |
| 河马 | 100 | 90.0 | 0.47 | カバ | 50 | 80.0 | 0.72 | Hippopotamus | 70 | 71.4 | 0.86 |
| 手提箱 | 100 | 90.0 | 0.47 | カバン | 100 | 80.0 | 0.72 | Suitcase | 100 | 50.0 | 0.50 |
| 南瓜 | 100 | 100.0 | 0.00 | カボチャ | 90 | 77.8 | 0.76 | Pumpkin | 100 | 100.0 | 0.00 |
| 独角仙 | 100 | 50.0 | 1.19 | カブトムシ | 90 | 66.7 | 0.92 | Beetle | 50 | 60.0 | 1.37 |
| 青蛙 | 100 | 100.0 | 0.00 | カエル | 100 | 80.0 | 0.72 | Frog | 90 | 100.0 | 0.00 |
| 镜子 | 100 | 80.0 | 0.92 | カガミ | 100 | 90.0 | 0.47 | Mirror | 100 | 100.0 | 0.00 |
| 钥匙 | 100 | 100.0 | 0.00 | カギ | 100 | 100.0 | 0.00 | Key | 100 | 100.0 | 0.00 |
| 锁 | 100 | 100.0 | 0.00 | カギ | 90 | 55.6 | 0.99 | Lock | 80 | 100.0 | 0.00 |
| 楼梯 | 100 | 100.0 | 0.00 | カイダン | 100 | 100.0 | 0.00 | Staircase | 100 | 90.0 | 0.47 |
| 乌龟 | 100 | 100.0 | 0.00 | カメ | 100 | 90.0 | 0.47 | Turtle | 60 | 100.0 | 0.00 |
| 袋鼠 | 100 | 100.0 | 0.00 | カンガルー | 90 | 100.0 | 0.00 | Kangaroo | 90 | 100.0 | 0.00 |
| 螃蟹 | 100 | 100.0 | 0.00 | カニ | 90 | 100.0 | 0.00 | Crab | 100 | 100.0 | 0.00 |
| 杯子 | 100 | 70.0 | 0.88 | カップ | 100 | 100.0 | 0.00 | Cup | 100 | 100.0 | 0.00 |
| 雨伞 | 100 | 100.0 | 0.00 | カサ | 100 | 100.0 | 0.00 | Umbrella | 100 | 100.0 | 0.00 |
| 武士刀 | 100 | 60.0 | 1.57 | カタナ | 100 | 60.0 | 0.91 | Japanese sword | 80 | 62.5 | 0.95 |
| 蜗牛 | 100 | 100.0 | 0.00 | カタツムリ | 20 | 100.0 | 0.00 | Snail | 50 | 100.0 | 0.00 |
| 窗帘 | 100 | 100.0 | 0.00 | カーテン | 90 | 100.0 | 0.00 | Curtain | 100 | 100.0 | 0.00 |
| 蛋糕 | 100 | 100.0 | 0.00 | ケーキ | 100 | 100.0 | 0.00 | Cake | 100 | 100.0 | 0.00 |
| 树 | 100 | 100.0 | 0.00 | キ | 100 | 100.0 | 0.00 | Tree | 100 | 100.0 | 0.00 |
| 金鱼 | 100 | 90.0 | 0.47 | キンギョ | 100 | 70.0 | 0.88 | Goldfish | 100 | 70.0 | 0.88 |
| 蘑菇 | 100 | 100.0 | 0.00 | キノコ | 100 | 100.0 | 0.00 | Mushroom | 100 | 90.0 | 0.47 |
| 长颈鹿 | 100 | 90.0 | 0.47 | キリン | 100 | 80.0 | 0.72 | Giraffe | 90 | 100.0 | 0.00 |
| 啄木鸟 | 100 | 100.0 | 0.00 | キツツキ | 100 | 100.0 | 0.00 | Woodpecker | 100 | 100.0 | 0.00 |
| 陀螺 | 100 | 100.0 | 0.00 | コマ | 10 | 100.0 | 0.00 | Top | 10 | 100.0 | 0.00 |
| 插头 | 100 | 70.0 | 1.16 | コンセント | 60 | 50.0 | 1.00 | Plug | 80 | 100.0 | 0.00 |
| 玻璃杯 | 100 | 80.0 | 0.92 | コップ | 100 | 80.0 | 0.72 | Glass | 100 | 80.0 | 0.92 |
| 外套 | 100 | 50.0 | 1.36 | コート | 100 | 100.0 | 0.00 | Coat | 100 | 100.0 | 0.00 |
| 蝙蝠 | 100 | 100.0 | 0.00 | コウモリ | 60 | 66.7 | 0.92 | Bat | 70 | 85.7 | 0.59 |
| 嘴唇 | 100 | 70.0 | 0.88 | クチビル | 100 | 100.0 | 0.00 | Lips | 100 | 70.0 | 0.88 |
| 钉子 | 100 | 90.0 | 0.47 | クギ | 70 | 71.4 | 0.86 | Nail | 50 | 80.0 | 0.72 |
| 孔雀 | 100 | 100.0 | 0.00 | クジャク | 80 | 75.0 | 0.81 | Peacock | 60 | 50.0 | 1.46 |
| 鲸鱼 | 100 | 80.0 | 0.59 | クジラ | 80 | 75.0 | 0.81 | Whale | 90 | 88.9 | 0.50 |
| 蜘蛛 | 100 | 90.0 | 0.47 | クモ | 90 | 88.9 | 0.50 | Spider | 100 | 90.0 | 0.47 |
| 云 | 100 | 90.0 | 0.47 | クモ | 100 | 90.0 | 0.47 | Cloud | 100 | 100.0 | 0.00 |
| 栗子 | 100 | 80.0 | 0.72 | クリ | 90 | 77.8 | 0.63 | Chestnut | 70 | 57.1 | 1.26 |
| 汽车 | 100 | 70.0 | 0.88 | クルマ | 100 | 100.0 | 0.00 | Car | 100 | 100.0 | 0.00 |
| 锁链 | 100 | 50.0 | 1.49 | クサリ | 70 | 85.7 | 0.59 | Chain | 90 | 88.9 | 0.50 |
| 梳子 | 100 | 100.0 | 0.00 | クシ | 70 | 71.4 | 0.86 | Comb | 70 | 57.1 | 0.99 |
| 鞋 | 100 | 70.0 | 0.88 | クツ | 100 | 100.0 | 0.00 | Shoe | 100 | 100.0 | 0.00 |
| 袜子 | 100 | 100.0 | 0.00 | クツシタ | 100 | 100.0 | 0.00 | Sock | 90 | 100.0 | 0.00 |
| 卷心菜 | 100 | 90.0 | 0.47 | キャベツ | 100 | 90.0 | 0.47 | Lettuce | 100 | 100.0 | 0.00 |
| 窗户 | 100 | 100.0 | 0.00 | マド | 100 | 100.0 | 0.00 | Window | 100 | 100.0 | 0.00 |
| 松树 | 100 | 60.0 | 1.57 | マツ | 100 | 100.0 | 0.00 | Japanese pine | 100 | 100.0 | 0.00 |
| 眼睛 | 100 | 100.0 | 0.00 | メ | 100 | 100.0 | 0.00 | Eye | 100 | 100.0 | 0.00 |
| 眼镜 | 100 | 100.0 | 0.00 | メガネ | 100 | 100.0 | 0.00 | Glasses | 100 | 100.0 | 0.00 |
| 甜瓜 | 100 | 60.0 | 1.30 | メロン | 100 | 90.0 | 0.47 | Melon | 100 | 100.0 | 0.00 |
| 月亮 | 100 | 100.0 | 0.00 | ミカヅキ | 100 | 100.0 | 0.00 | Moon | 100 | 100.0 | 0.00 |
| 橘子 | 100 | 70.0 | 1.16 | ミカン | 100 | 60.0 | 0.97 | Orange | 100 | 100.0 | 0.00 |
| 耳朵 | 100 | 100.0 | 0.00 | ミミ | 100 | 100.0 | 0.00 | Ear | 100 | 100.0 | 0.00 |
| 缝纫机 | 100 | 100.0 | 0.00 | ミシン | 10 | 100.0 | 0.00 | Sewing machine | 50 | 60.0 | 0.97 |
| 桃子 | 100 | 90.0 | 0.47 | モモ | 100 | 100.0 | 0.00 | Peach | 100 | 100.0 | 0.00 |
| 猫 | 100 | 100.0 | 0.00 | ネコ | 100 | 100.0 | 0.00 | Cat | 100 | 100.0 | 0.00 |
| 项链 | 100 | 100.0 | 0.00 | ネックレス | 100 | 100.0 | 0.00 | Necklace | 100 | 100.0 | 0.00 |
| 领带 | 100 | 100.0 | 0.00 | ネクタイ | 100 | 70.0 | 0.88 | Tie | 100 | 100.0 | 0.00 |
| 老鼠 | 100 | 100.0 | 0.00 | ネズミ | 100 | 100.0 | 0.00 | Mouse | 100 | 70.0 | 0.88 |
| 胡萝卜 | 100 | 60.0 | 0.97 | ニンジン | 100 | 100.0 | 0.00 | Carrot | 100 | 100.0 | 0.00 |
| 鸡 | 100 | 60.0 | 0.97 | ニワトリ | 100 | 70.0 | 0.88 | Chicken | 100 | 60.0 | 1.37 |
| 锯子 | 100 | 90.0 | 0.47 | ノコギリ | 50 | 60.0 | 0.91 | Saw | 80 | 62.5 | 0.95 |
| 斧头 | 100 | 80.0 | 0.72 | オノ | 50 | 80.0 | 0.72 | Axe | 60 | 66.7 | 1.25 |
| 狼 | 100 | 60.0 | 0.97 | オオカミ | 100 | 70.0 | 0.88 | Wolf | 90 | 88.9 | 0.50 |
| 风琴 | 100 | 100.0 | 0.00 | オルガン | 100 | 100.0 | 0.00 | Choir organ | 100 | 100.0 | 0.00 |
| 日本城堡 | 100 | 70.0 | 1.02 | シロ（日本） | 100 | 80.0 | 0.72 | Japanese castle | 100 | 80.0 | 0.72 |
| 皇冠 | 100 | 90.0 | 0.47 | オウカン | 70 | 100.0 | 0.00 | Crown | 80 | 87.5 | 0.54 |
| 菠萝 | 100 | 100.0 | 0.33 | パイナップル | 100 | 100.0 | 0.00 | Pineapple | 100 | 100.0 | 0.00 |
| 烟斗 | 100 | 90.0 | 0.47 | パイプ | 70 | 57.1 | 0.99 | Pipe | 60 | 83.3 | 0.65 |
| 面包 | 100 | 90.0 | 0.47 | パン | 100 | 100.0 | 0.00 | Bread | 100 | 100.0 | 0.00 |
| 钳子 | 100 | 80.0 | 0.92 | ペンチ | 50 | 60.0 | 0.97 | Pliers | 10 | 100.0 | 0.00 |
| 企鹅 | 100 | 100.0 | 0.00 | ペンギン | 100 | 90.0 | 0.47 | Penguin | 100 | 100.0 | 0.00 |
| 鹈鹕 | 100 | 70.0 | 1.36 | ペリカン | 90 | 88.9 | 0.50 | Pelican | 100 | 100.0 | 0.00 |
| 钢琴 | 100 | 90.0 | 0.47 | グランドピアノ | 100 | 100.0 | 0.00 | Piano | 100 | 100.0 | 0.00 |
| 青椒 | 100 | 80.0 | 0.92 | ピーマン | 70 | 100.0 | 0.00 | Pepper | 60 | 100.0 | 0.00 |
| 花生 | 100 | 100.0 | 0.00 | ピーナッツ | 80 | 100.0 | 0.00 | Peanut | 90 | 100.0 | 0.00 |
| 金字塔 | 100 | 100.0 | 0.00 | ピラミッド | 80 | 100.0 | 0.00 | Pyramid | 60 | 100.0 | 0.00 |
| 手枪 | 100 | 70.0 | 1.16 | ピストル | 90 | 77.8 | 0.76 | Gun | 100 | 90.0 | 0.47 |
| 游泳池 | 100 | 100.0 | 0.00 | プール | 100 | 100.0 | 0.00 | Swimming pool | 100 | 100.0 | 0.00 |
| 狮子 | 100 | 100.0 | 0.00 | ライオン | 100 | 100.0 | 0.00 | Lion | 100 | 100.0 | 0.00 |
| 收音机 | 100 | 100.0 | 0.00 | ラジオ | 80 | 100.0 | 0.00 | Radio | 90 | 88.9 | 0.63 |
| 网球拍 | 100 | 70.0 | 0.88 | ラケット | 70 | 57.1 | 0.98 | Tennis racket | 70 | 42.9 | 1.56 |
| 骆驼 | 100 | 100.0 | 0.00 | ラクダ | 80 | 75.0 | 0.81 | Camel | 70 | 85.7 | 0.59 |
| 冰箱 | 100 | 100.0 | 0.00 | レイゾウコ | 100 | 100.0 | 0.00 | Refrigerator | 100 | 100.0 | 0.00 |
| 柠檬 | 100 | 100.0 | 0.00 | レモン | 100 | 100.0 | 0.00 | Lemon | 100 | 100.0 | 0.00 |
| 蝴蝶结 | 100 | 100.0 | 0.00 | リボン | 50 | 100.0 | 0.00 | Bow | 50 | 100.0 | 0.00 |
| 苹果 | 100 | 100.0 | 0.00 | リンゴ | 100 | 100.0 | 0.00 | Apple | 100 | 100.0 | 0.00 |
| 松鼠 | 100 | 60.0 | 1.37 | リス | 90 | 55.6 | 0.99 | Squirrel | 90 | 66.7 | 1.22 |
| 火箭 | 100 | 100.0 | 0.00 | ロケット | 100 | 100.0 | 0.00 | Rocket | 100 | 100.0 | 0.00 |
| 蜡烛 | 100 | 100.0 | 0.00 | ロウソク | 90 | 66.7 | 0.92 | Candle | 90 | 100.0 | 0.00 |
| 鱼 | 100 | 100.0 | 0.00 | サカナ | 100 | 100.0 | 0.00 | Fish | 100 | 100.0 | 0.00 |
| 栅栏 | 100 | 100.0 | 0.00 | サク | 30 | 66.7 | 0.92 | Fence | 50 | 100.0 | 0.00 |
| 樱花 | 100 | 60.0 | 1.37 | サクラ | 100 | 90.0 | 0.47 | Cherry blossom | 100 | 70.0 | 0.88 |
| 樱桃 | 100 | 90.0 | 0.47 | サクランボ | 90 | 55.6 | 0.99 | Cherry | 100 | 80.0 | 0.92 |
| 鲨鱼 | 100 | 90.0 | 0.47 | サメ | 100 | 90.0 | 0.47 | Shark | 100 | 100.0 | 0.00 |
| 三明治 | 100 | 100.0 | 0.00 | サンドイッチ | 100 | 100.0 | 0.00 | Sandwich | 100 | 100.0 | 0.00 |
| 三角尺 | 100 | 70.0 | 1.16 | サンカクジョウギ | 70 | 71.4 | 0.86 | Triangle ruler | 100 | 90.0 | 0.47 |
| 三轮车 | 100 | 90.0 | 0.47 | サンリンシャ | 100 | 60.0 | 0.97 | Tricycle | 90 | 66.7 | 0.92 |
| 盘子 | 100 | 100.0 | 0.33 | サラ | 100 | 90.0 | 0.47 | Plate | 100 | 100.0 | 0.00 |
| 猴子 | 100 | 90.0 | 0.47 | サル | 90 | 100.0 | 0.00 | Monkey | 100 | 100.0 | 0.00 |
| 坦克 | 100 | 100.0 | 0.00 | センシャ | 100 | 70.0 | 0.88 | Tank | 100 | 100.0 | 0.00 |
| 毛衣 | 100 | 100.0 | 0.00 | セーター | 70 | 57.1 | 0.99 | Sweater | 90 | 88.9 | 0.50 |
| 车轮 | 100 | 100.0 | 0.00 | シャリン | 80 | 87.5 | 0.54 | Wheel | 100 | 70.0 | 1.16 |
| 衬衫 | 100 | 80.0 | 0.92 | ワイシャツ | 100 | 90.0 | 0.47 | Shirt | 100 | 90.0 | 0.47 |
| 鹿 | 100 | 100.0 | 0.00 | シカ | 100 | 100.0 | 0.00 | Deer | 100 | 90.0 | 0.47 |
| 斑马 | 100 | 100.0 | 0.00 | シマウマ | 90 | 66.7 | 1.22 | Zebra | 90 | 88.9 | 0.50 |
| 红绿灯 | 100 | 70.0 | 0.88 | シンゴウ | 100 | 100.0 | 0.00 | Traffic light | 80 | 62.5 | 0.95 |
| 城堡 | 100 | 100.0 | 0.00 | シロ（西洋） | 90 | 100.0 | 0.00 | Castle | 100 | 100.0 | 0.00 |
| 熊 | 100 | 70.0 | 1.16 | シロクマ | 100 | 100.0 | 0.00 | Bear | 100 | 100.0 | 0.00 |
| 舌头 | 100 | 100.0 | 0.00 | シタ | 100 | 80.0 | 0.72 | Tongue | 100 | 100.0 | 0.00 |
| 沙发 | 100 | 100.0 | 0.00 | ソファー | 100 | 100.0 | 0.00 | Couch | 100 | 100.0 | 0.00 |
| 开关 | 100 | 100.0 | 0.00 | スイッチ | 100 | 90.0 | 0.47 | Light switch | 100 | 100.0 | 0.00 |
| 裙子 | 100 | 80.0 | 0.46 | スカート | 100 | 100.0 | 0.00 | Skirt | 100 | 100.0 | 0.00 |
| 滑冰鞋 | 100 | 100.0 | 0.00 | スケートグツ | 80 | 87.5 | 0.54 | Skating boots | 100 | 70.0 | 0.88 |
| 勺子 | 100 | 100.0 | 0.00 | スプーン | 100 | 100.0 | 0.00 | Spoon | 100 | 100.0 | 0.00 |
| 拖鞋 | 100 | 100.0 | 0.00 | スリッパ | 100 | 90.0 | 0.47 | Slippers | 100 | 100.0 | 0.00 |
| 香烟 | 100 | 100.0 | 0.00 | タバコ | 100 | 100.0 | 0.00 | Cigarette | 100 | 50.0 | 1.02 |
| 鼓 | 80 | 75.0 | 0.81 | タイコ | 60 | 66.7 | 0.92 | Drum | 80 | 87.5 | 0.54 |
| 太阳 | 100 | 100.0 | 0.00 | タイヨウ | 100 | 100.0 | 0.00 | Sun | 100 | 100.0 | 0.00 |
| 竹子 | 100 | 100.0 | 0.00 | タケ | 100 | 100.0 | 0.00 | Bamboo | 90 | 100.0 | 0.00 |
| 瀑布 | 100 | 90.0 | 0.47 | タキ | 80 | 100.0 | 0.00 | Waterfall | 60 | 83.3 | 0.65 |
| 风筝 | 100 | 100.0 | 0.33 | タコ | 30 | 66.7 | 0.92 | Kite | 80 | 100.0 | 0.00 |
| 洋葱 | 100 | 80.0 | 0.92 | タマネギ | 90 | 88.9 | 0.50 | Onion | 80 | 100.0 | 0.00 |
| 抽屉柜 | 100 | 80.0 | 0.92 | タンス | 60 | 33.3 | 1.92 | Dresser | 80 | 50.0 | 1.75 |
| 木桶 | 100 | 80.0 | 0.92 | タル | 30 | 66.7 | 0.92 | Barrel | 60 | 100.0 | 0.00 |
| 手 | 100 | 100.0 | 0.00 | テ | 100 | 100.0 | 0.00 | Hand | 100 | 100.0 | 0.00 |
| 手套 | 100 | 100.0 | 0.00 | テブクロ | 100 | 100.0 | 0.00 | Mitten | 100 | 100.0 | 0.00 |
| 电视 | 100 | 100.0 | 0.00 | テレビ | 100 | 100.0 | 0.00 | Television | 100 | 100.0 | 0.00 |
| 帐篷 | 100 | 100.0 | 0.00 | テント | 80 | 100.0 | 0.00 | Tent | 90 | 77.8 | 0.76 |
| 瓢虫 | 100 | 80.0 | 0.72 | テントウムシ | 100 | 80.0 | 0.72 | Ladybug | 60 | 50.0 | 1.00 |
| T恤 | 100 | 70.0 | 1.36 | ティーシャツ | 100 | 80.0 | 0.72 | T-Shirt | 100 | 90.0 | 0.47 |
| 时钟 | 100 | 70.0 | 1.16 | トケイ | 100 | 100.0 | 0.00 | Clock | 90 | 100.0 | 0.00 |
| 番茄 | 100 | 100.0 | 0.00 | トマト | 100 | 100.0 | 0.00 | Tomato | 100 | 100.0 | 0.00 |
| 蜻蜓 | 100 | 100.0 | 0.00 | トンボ | 60 | 83.3 | 0.65 | Dragonfly | 50 | 60.0 | 1.37 |
| 老虎 | 100 | 90.0 | 0.47 | トラ | 100 | 80.0 | 0.72 | Tiger | 100 | 100.0 | 0.00 |
| 卡车 | 100 | 100.0 | 0.00 | トラック | 100 | 70.0 | 0.88 | Truck | 100 | 80.0 | 0.72 |
| 扑克牌 | 100 | 90.0 | 0.47 | トランプ | 100 | 70.0 | 0.88 | Playing cards | 100 | 60.0 | 0.97 |
| 烤面包机 | 100 | 60.0 | 1.30 | トースター | 50 | 60.0 | 0.97 | Toaster | 80 | 62.5 | 1.30 |
| 玉米 | 100 | 100.0 | 0.00 | トウモロコシ | 100 | 90.0 | 0.47 | Corn | 90 | 100.0 | 0.00 |
| 桌子 | 100 | 100.0 | 0.00 | ツクエ | 100 | 70.0 | 0.88 | Table | 100 | 80.0 | 0.72 |
| 郁金香 | 100 | 70.0 | 1.16 | チューリップ | 100 | 70.0 | 0.88 | Tulip | 100 | 60.0 | 1.30 |
| 手表 | 100 | 100.0 | 0.00 | ウデドケイ | 100 | 100.0 | 0.00 | Watch | 100 | 100.0 | 0.00 |
| 马 | 100 | 100.0 | 0.00 | ウマ | 100 | 100.0 | 0.00 | Horse | 100 | 100.0 | 0.00 |
| 鳗鱼 | 100 | 90.0 | 0.14 | ウナギ | 100 | 90.0 | 0.47 | Eel | 80 | 50.0 | 1.00 |
| 兔子 | 100 | 100.0 | 0.00 | ウサギ | 100 | 90.0 | 0.47 | Rabbit | 100 | 100.0 | 0.00 |
| 牛 | 100 | 100.0 | 0.00 | ウシ | 100 | 90.0 | 0.47 | Cow | 100 | 100.0 | 0.00 |
| 小提琴 | 100 | 100.0 | 0.00 | バイオリン | 100 | 90.0 | 0.47 | Violin | 100 | 100.0 | 0.00 |
| 鳄鱼 | 100 | 100.0 | 0.00 | ワニ | 70 | 57.1 | 0.99 | Alligator | 70 | 100.0 | 0.00 |
| 山羊 | 100 | 80.0 | 0.92 | ヤギ | 100 | 50.0 | 1.03 | Goat | 90 | 88.9 | 0.50 |
| 箭头 | 100 | 90.0 | 0.47 | ヤジルシ | 80 | 87.5 | 0.54 | Arrow | 90 | 100.0 | 0.00 |
| 烧水壶 | 100 | 90.0 | 0.47 | ヤカン | 80 | 50.0 | 1.00 | Kettle | 70 | 57.1 | 0.99 |
| 山 | 100 | 100.0 | 0.00 | ヤマ | 100 | 100.0 | 0.00 | Mountain | 100 | 100.0 | 0.00 |
| 梨 | 100 | 100.0 | 0.00 | ヨウナシ | 100 | 80.0 | 0.72 | Pear | 100 | 80.0 | 0.72 |
| 戒指 | 100 | 100.0 | 0.00 | ユビワ | 100 | 90.0 | 0.47 | Ring | 100 | 100.0 | 0.00 |
| 雪人 | 100 | 100.0 | 0.00 | ユキダルマ | 100 | 100.0 | 0.00 | Snowman | 100 | 90.0 | 0.47 |
| 百合花 | 100 | 90.0 | 0.47 | ユリ | 100 | 60.0 | 0.97 | Lily | 100 | 60.0 | 0.97 |
| 大象 | 100 | 100.0 | 0.00 | ゾウ | 100 | 100.0 | 0.00 | Elephant | 100 | 100.0 | 0.00 |
| 裤子 | 100 | 100.0 | 0.00 | ズボン | 90 | 77.8 | 0.76 | Pants | 90 | 77.8 | 0.76 |
| Mean | 99.8 | 89.5 | 0.38 |  | 87.7 | 87.2 | 0.39 |  | 88.3 | 90.1 | 0.31 |

**Table S6. Comparison between Pearson and partial correlation analyses**

Note: AoA: age of acquisition
FG: fusiform gyrus; PITG: posterior inferior temporal gyrus; PT: pars triangularis; PO: pars opercularis; SPL: superior parietal lobe; SMG: supra marginal gyrus; ACG: anterior cingulate gyrus; CN: caudate nucleus.

| Outcome | Measure | Pearson correlation | | Partial correlation | |
| --- | --- | --- | --- | --- | --- |
|  |  | r | p | r | p |
| **Japanese language** |  |  |  |  |  |
|  | AoA vs Proficiency | 0.21 | 0.54 |  |  |
| Behavioral measure |  |  |  |  |  |
|  | AoA vs RT | -0.29 | 0.40 | -0.22 | 0.55 |
|  | Proficiency vs RT | -0.48 | 0.14 | -0.44 | 0.20 |
|  | AoA vs Accuracy | 0.10 | 0.77 | 0.03 | 0.94 |
|  | Proficiency vs Accuracy | 0.36 | 0.27 | 0.35 | 0.32 |
| Neural indices |  |  |  |  |  |
| Activation (LG) | AoA vs Signal change (%) | -0.17 | 0.62 | -0.12 | 0.74 |
|  | Proficiency vs Signal change (%) | -0.26 | 0.43 | -0.24 | 0.51 |
| Activation (MOG) | AoA vs Signal change (%) | 0.12 | 0.73 | -0.04 | 0.91 |
|  | **Proficiency vs Signal change (%)** | **0.71** | **0.01** | **0.71** | **0.02** |
| **VOI-based RSA CN-JA similarity** |  |  |  |  |  |
| FG | AoA | 0.32 | 0.34 | 0.30 | 0.40 |
|  | Proficiency | 0.14 | 0.69 | 0.08 | 0.83 |
| PITG | AoA | 0.16 | 0.63 | 0.13 | 0.71 |
|  | Proficiency | 0.18 | 0.60 | 0.15 | 0.68 |
| PT | AoA | 0.16 | 0.64 | 0.07 | 0.85 |
|  | Proficiency | **0.49** | **0.12** | **0.47** | **0.17** |
| PO | AoA | -0.22 | 0.52 | -0.32 | 0.37 |
|  | Proficiency | 0.35 | 0.28 | 0.42 | 0.23 |
| SMG | AoA | 0.44 | 0.18 | 0.40 | 0.25 |
|  | Proficiency | 0.30 | 0.36 | 0.25 | 0.49 |
| SPL | AoA | -0.22 | 0.51 | -0.28 | 0.43 |
|  | Proficiency | 0.22 | 0.50 | 0.28 | 0.43 |
| ACC | AoA | 0.37 | 0.12 | 0.38 | 0.13 |
|  | Proficiency | **0.53** | **0.09** | **0.54** | **0.11** |
| Caudate | AoA | -0.05 | 0.88 | -0.16 | 0.67 |
|  | Proficiency | 0.42 | 0.19 | 0.45 | 0.19 |
| **English language** |  |  |  |  |  |
|  | AoA vs Proficiency | 0.02 | 0.94 |  |  |
| Behavioral measure |  |  |  |  |  |
|  | **AoA vs RT** | **0.60** | **0.02** | **0.61** | **0.02** |
|  | Proficiency vs RT | 0.16 | 0.56 | 0.19 | 0.50 |
|  | AoA vs Accuracy | -0.37 | 0.17 | -0.38 | 0.16 |
|  | Proficiency vs Accuracy | 0.17 | 0.55 | 0.19 | 0.50 |
| **VOI-based RSA**  **CN-EN similarity** |  |  |  |  |  |
| FG | AoA | 0.35 | 0.19 | 0.35 | 0.19 |
|  | Proficiency | 0.05 | 0.85 | 0.05 | 0.87 |
| PITG | AoA | 0.32 | 0.25 | 0.33 | 0.25 |
|  | Proficiency | 0.09 | 0.72 | 0.10 | 0.72 |
| PT | AoA | 0.26 | 0.38 | 0.27 | 0.38 |
|  | Proficiency | -0.01 | 0.98 | -0.02 | 0.94 |
| PO | AoA | 0.19 | 0.27 | 0.19 | 0.26 |
|  | Proficiency | 0.16 | 0.58 | 0.17 | 0.56 |
| SMG | AoA | 0.21 | 0.45 | 0.21 | 0.45 |
|  | Proficiency | 0.11 | 0.70 | 0.11 | 0.71 |
| SPL | AoA | 0.28 | 0.31 | 0.29 | 0.29 |
|  | Proficiency | 0.35 | 0.19 | 0.36 | 0.18 |
| ACC | AoA | 0.34 | 0.21 | 0.35 | 0.20 |
|  | Proficiency | -0.16 | 0.57 | -0.18 | 0.52 |
| Caudate | AoA | 0.31 | 0.26 | 0.34 | 0.22 |
|  | Proficiency | -0.35 | 0.20 | -0.38 | 0.17 |

**
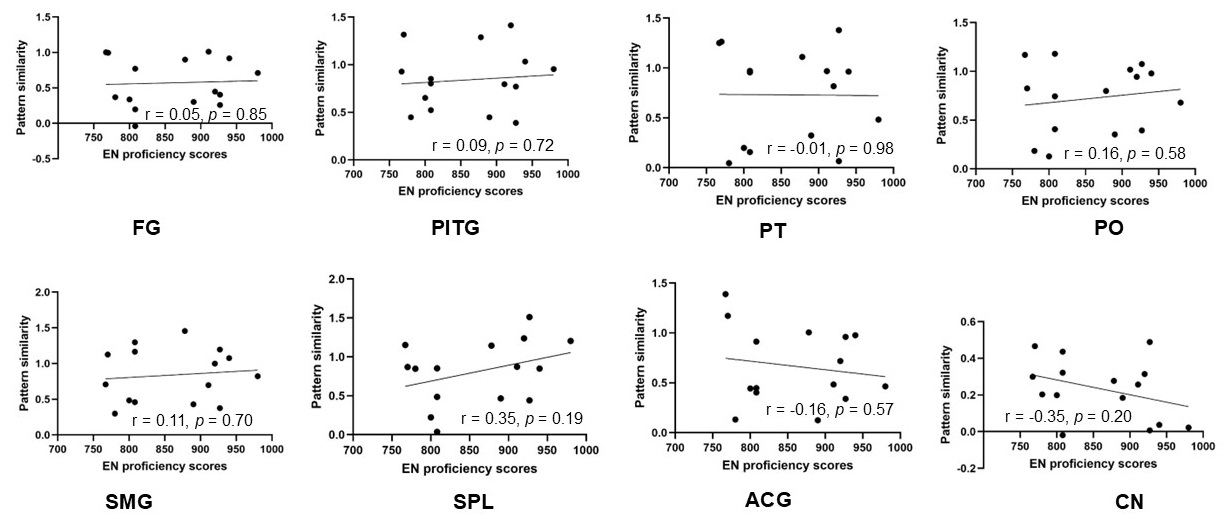
**

**Figure S1. Correlation between ROI-based RSA of Chinese–English conditions and English proficiency**No significant correlation was found. FG: fusiform gyrus; PITG: posterior inferior temporal gyrus; PT: pars triangularis; PO: pars opercularis; SMG: supra marginal gyrus; SPL: superior parietal lobe; ACG: anterior cingulate gyrus; CN: caudate nucleus.
